# Triggering Dectin-1-Pathway Alone Is Not Sufficient to Induce Cytokine Production by Murine Macrophages

**DOI:** 10.1371/journal.pone.0148464

**Published:** 2016-02-03

**Authors:** Sarah Walachowski, Guillaume Tabouret, Gilles Foucras

**Affiliations:** 1 Université de Toulouse, INP-ENVT, UMR 1225, IHAP, Toulouse, France; 2 INRA, UMR1225, IHAP, Toulouse, France; Friedrich-Alexander-University Erlangen, GERMANY

## Abstract

β-glucans (BG) are abundant polysaccharides of the *Saccharomyces cerevisiae* cell wall (*Sc* CW), an industry byproduct. They have immuno-stimulatory properties upon engagement of dectin-1 (*Clec7a)*, their main receptor on particular immune cells, and they actually become of great interest because of their preventive or therapeutic potentials. Zymosan, a crude extract of *Sc* CW was studied as a prototypic BG, despite its miscellaneous PAMPs content. Here, we examined the response of murine wild type or *Clec7a*^-/-^ bone marrow-derived macrophages (BMDM) to products with increasing BG content (15, 65 or 75%) and compared their effects with those of other dectin-1 ligands. The enrichment process removed TLR ligands while preserving dectin-1 activity. The most enriched extracts have very low NFκB activity and triggered low amounts of cytokine production in contrast with crude products like zymosan and BG15. Furthermore, *MyD88*^-/-^ BMDM did not produce TNFα in response to crude *Sc* CW extracts, whereas their response to BG-enriched extracts was unaffected, suggesting that BG alone are not able to initiate cytokine secretion. Although *Sc* CW-derived BG stimulated the late and strong expression of *Csf2* in a dectin-1-dependent manner, they remain poor inducers of chemokine and cytokine production in murine macrophages.

## Introduction

For centuries, fungal cell wall components have been commonly used in traditional Oriental medicine [1], and their benefits are mainly attributed to β-glucans (BG). These polysaccharides have recently become of interest in Western countries, because of their activity on innate immune cells and their potential therapeutic applications. Indeed, BG have various valuable properties including antitumor [2], antidiabetic, and anti-microbial activity against viral, bacterial and fungal diseases (reviewed by *Chen et al* [3]). Strikingly, Lentinan has been shown to improve the survival rate of Japanese patients with gastric and colorectal cancers [4]. Given the various potential health benefits attributed to BG, many dietary supplements containing BG are available. BG are the most abundant polysaccharides of the fungal cell wall. They are intermingled with chitins (polymers of N-acetylglucosamine) that are located near the plasma membrane, and mannans that are anchored to the outer cell wall, all of which are interspersed with glycoproteins [5]. β-glucans are polymers of glucose and have a backbone of linear β(1,3)-linked D-glucose molecules with β(1,6)-linked side chains of diverse sizes occurring at different intervals along the backbone. Although BG can be extracted from mushrooms, oat, barley, seaweed, algae, bacteria and yeast, the composition of the cell wall varies greatly within and between species and the length and types of linkage also differ [6,7]. This heterogeneity of structure, conformation, source and even nature (soluble or particulate) among BG influences the biological response of immune cells. This may explain conflicting findings regarding the effects of BG in human or animal models. Moreover, the effects of one preparation of ‘β-glucans’ are often inappropriately extrapolated to all β-glucans, and ‘β-glucans’ need to be characterized to better define the type of immune response that they elicit. Indeed, the total BG-content of extracts used for investigation is rarely determined. Despite the complexity of BG, several molecules [8,9] including complement receptor 3 (CR3), lactosylceramide (LacCer), scavenger receptor (SRs) and dectin-1 [10,11], recognize BG on the cell surface of macrophages, monocytes, dendritic cells, NK cells and neutrophils [12].

So far, most studies designed to investigate the activities of BG have used zymosan [13], which is considered as a source of BG. However, the BG content of zymosan rarely exceeds 50% [2,14], which is similar to the BG content of the *Sc* CW [15,16]. Other components are also present that are PAMPs for several other PRRs including TLRs on innate immune cells. *Goodridge et al* recently demonstrated that zymosan particles strongly stimulate macrophages to produce TNFα, as a result of dectin-1, which activates NFκB [17,18]. Moreover, dectin-1 and TLR2 work together to enhance TLR2-mediated NFκB signaling and the release of pro-inflammatory cytokines [19]. Nevertheless, it remains unclear how dectin-1 interacts with *Sc* BG compounds. The main challenging issue of this study was to determine whether dectin-1 is involved to the macrophage response triggered by a series of three increasingly BG-enriched CW extracted from the same strain of *Saccharomyces cerevisiae*. Contrary to conventional BG wisdom, we found that *Sc* CW extracts highly-enriched in BG do not elicit NFκB activity and that dectin-1-dependent-cytokine production in response to these compounds is very much lower than that induced by crude compounds like zymosan. These unpurified products have the capacity to induce TLR2 and TLR4 activities resulting in strong signaling via MyD88 and NFκB. Therefore, our data show that the robust response elicited by zymosan is not strictly caused by its BG content. Altogether our findings demonstrate that BG are poor inducers of cytokine production in murine macrophages.

## Materials and Methods

### Reagents

RPMI 1640 GlutaMAX^™^, DMEM GlutaMAX^™^, PBS, Non-Essential Amino Acids (NEAA), sodium pyruvate and antibiotics for cell culture such as Penicillin-Streptomycin (Pen-Strep^™^), Gentamicin^™^, Normocin^™^ and Zeocin^™^ were purchased from GIBCO (Life Technologies). Fetal Bovine Serum (FBS) was provided by Eurobio, France. The dectin-1 and TLR ligands used as controls were from InvivoGen. The following compounds were compared with *Sc* BG compounds as described below: zymosan (zym), particulate (WGPd) and soluble glucan-enriched compound (WGPs) and curdlan (curd), a linear β1,3-glucan extracted from *Alcaligenes faecalis*. The synthetic triacylated lipoprotein Pam3CSK4 and ultraPure lipopolysaccharide (LPS) from E. coli O111:B4 both purchased from InvivoGen (France).

BG compounds of interest were extracted from the same strain of *Saccharomyces cerevisiae* (*Sc*) owned by Phileo-Lesaffre Animal Care. The crude extract originally contained a total BG content of 15% (BG15) and was further enriched using several methods to generate two compounds, BG65 and BG75, with a total BG content of 65% and 75%, respectively. The BG15 extraction method from *S*. *cerevisiae* was mainly the same as for zymosan preparation [20] and resulted in “ghost” cells with an average particle diameter of 3 μm deprived of nucleic acid [14]. BG65 was obtained from a suspension of BG15 (14%) by hot alkaline hydrolysis (4% NaOH, 90°C, 2 h) to remove mannoproteins and lipids. BG75, the most enriched *Sc* CW, was prepared from a suspension of BG65 (14%) which was submitted to an enzymatic hydrolysis (amylase and amyloglucosidase) to eliminate the contaminant glycogen. Both products were then lyophilized (dry matter > 94%). The BG content of each *Sc* CW preparation was determined using the enzymatic yeast beta-glucan kit (K-EBHLG, Megazyme, Ireland) and the total amount of glucose released was measured by HPLC. Dry powders were suspended in cell culture medium at 10 mg/mL and were then dispersed by pipetting 20 times up and down through a syringe fitted with a 26G needle to optimize bioavailability. Composition of BG extracts is presented in Table 1. According to the manufacturer, WGPd and curdlan used in this study contain more than 75% and 80% of BG, respectively. Concerning zymosan, the content in BG did not differ from a yeast cell wall and is comprised between 50 and 57% (percentage of dry matter) [14].

**Table 1:** Main composition of the *Saccharomyces cerevisiae* cell wall compounds.

	Yeast Cell Wall Preparation		
Constituents	BG15	BG65	BG75
**Dry matter (%)**	94.3	95.8	94.0
**Beta-glucans (%)**	16.3	67.1	77.0
**Glycogen (%)**	9.4	13.0	0.0
**Mannans (%)**	18.9	3.7	0.7
**Total glucans (%)**	25.7	69.1	74.4
**Proteins (%)**	33.3	3.4	3.6

### Culture of reporter cell lines

The NFκB reporter cell lines HEK-Blue^™^-hTLR2, HEK-Blue^™^-hTLR4, HEK-Blue^™^-hTLR9 and HEK-Blue^™^ -hDectin-1 (InvivoGen) which overexpress the human TLR2, TLR4, TLR9 or dectin-1 receptors and the Secreted Embryonic Alkaline Phosphatase (SEAP) reporter gene driven by an NFκB-inducible promoter. A recombinant HEK293 cell line expressing the reporter gene only (HEK-Blue^™^-null) was used as a negative control.

RAW-Blue^™^ Cells (InvivoGen) are derived from murine RAW264.7 macrophages containing a chromosomal integration of an SEAP reporter construct driven by NFκB and AP-1.

All cell lines were cultured and propagated according to the manufacturer’s recommendations. Briefly, HEK-Blue^™^ and RAW-Blue^™^ cells were grown in medium containing high glucose DMEM GlutaMAX^™^ supplemented with 10% heat-inactivated FBS (30 min, 56°C), 100 U/mL Penicillin-Streptomycin, 100 μg/mL Normocin^™^ and appropriate selective antibiotics.

### Measurement of TLR, dectin-1 and NFκB activity using reporter cell lines

For HEK-Blue-hTLR and -hdectin1 assays, cells were plated in 96-well tissue culture plates at 1x10^5^ cells/well in HEK-Blue^™^ Detection medium containing the substrate for SEAP. Serial dilutions of *Sc* BG and other sourced BG extracts were used for stimulation Supernatants were transferred to a new plate and the optical density (OD 650 nm) was measured (VERSAmax plate reader, Molecular Devices).

To assess NFκB activity, RAW-Blue^™^ macrophages were cultured in the recommended medium and incubated in the same conditions. SEAP was measured using a colorimetric enzymatic assay. Supernatants were incubated with Quanti-Blue^™^ (InvivoGen) 25% v/v. for 2 h at 37°C, and OD 650nm was measured.

### Immortalized BMDM culture and functional assay

Murine wild type (WT) and *MyD88*^-/-^ immortalized BMDM (iBMDM) were generously provided by Dr. R. Volmer (IHAP, Toulouse), to examine the involvement of TLR pathways in the cellular response to *Sc* BG compounds (BG15, BG65 and BG75) and controls (zymosan and WGPd). These cells were subcultured in high glucose DMEM GlutaMAX^™^ supplemented with 10% heat-inactivated FBS (30 min, 56°C) and 100 U/mL Pen-Strep. Cells were plated in 96-well plates at 1x10^5^ iBMDM/well for 16 h until complete adherence before being stimulated with *Sc* BG compounds and controls. Supernatants were collected and stored at -20°C until further use.

### Animals

WT C57Bl/6 and DBA/2 mice were purchased from Janvier Labs (St Berthevin, France) and *Clec7a*^-/-^ mice [11] were originally provided by Pr. Gordon Brown (University of Aberdeen, Scotland) and were bred in-house. Eight to 12-week old C57Bl/6 *Clec7a*^-/-^ mice and their strain-matched WT controls from both sexes, and female DBA/2 mice were housed under pathogen-free conditions in an accredited research animal facility of the National Veterinary College (UMR IHAP, Toulouse, France). This study was carried out in strict accordance with the Federation of European Laboratory Animal Science Association guidelines (FELASA). Experiments were performed by FELASA accredited investigators (n° 311155580) in strict accordance with the recommendations of the local ethics committee, ‘Science et Santé Animale’ (SSA). Mice were sacrificed by cervical dislocation and all efforts were made to minimize animal pain and distress.

### Peritoneal cells collection and flow cytometry analysis

Inflammatory peritoneal macrophages were elicited by intra-peritoneal injection for 4 days of sterile 4% brewer thioglycollate medium (Fluka Analytical, Sigma-Aldrich) prepared according to the manufacturer’s recommendations. A model of peritonitis was used to evaluate the inflammatory response intensity induced by our BG compounds. Mice were challenged for 6 h with 100 μg/mice of BG15, BG75 and zymosan. Total peritoneal cells including inflammatory monocytes, neutrophils and macrophages (resident peritoneal macrophages (RPM) or thioglycollate-elicited macrophages (TEPM)) were harvested from the peritoneal cavities of mice by lavage with cold PBS supplemented with 5mM EDTA and 0.2% heat-inactivated FBS. After collection, cells were centrifuged (300g, 5 minutes) and absolute cells and macrophages numbers were determined by flow cytometry (MACSQuant^®^, Miltenyi Biotech, Germany). Cells were preincubated with anti-CD16/CD32 (Biolegend, Ozyme-France) to block FcγRII/III receptors and then incubated with the following fluorochrome conjugated mAbs: anti-CD115 (CSF-1R; Biolegend, Ozyme-France) and anti-dectin-1 (2A11; AbD serotec) or its isotype control IgG1 (A110-1, BD biosciences Pharmingen). Anti-Ly6G mAb (1A8, Biolegend) or combination of anti-F4-80 (CI:A3-1; AbD serotec) and anti-Ly6C (HK1.4, Biolegend) mAbs labeling were used to identify neutrophils and inflammatory monocytes, respectively. A 7-AAD staining (Biolegend, Ozyme-France) was used to discriminate death cells and doublets of cells were excluded with a gating on FSC-H/FSC-A. The acquisition was performed on 1x10^5^ cells using MACSQuantify software (Miltenyi Biotech, Germany). Data were analyzed with FlowJo software (FlowJo LLC, USA).

### Primary cell culture and functional assays

Murine BMDM were obtained as described previously [21]. Briefly, myeloid cells precursors from bone marrow were matured for 7 days (37°C, 5% CO_2_) in complete RPMI (containing RPMI GlutaMAX^™^ supplemented with 10%, heat-inactivated FBS, 1% sodium pyruvate, 1% NEAA, 100 U/mL Pen-Strep or 50 μg/mL gentamicin) supplemented with 30% LADMAC (ATCC^®^ CRL-2420^™^ [22,23]) cell-conditioned medium (LCM) as a source of M-CSF. After differentiation, the cell preparation contained a homogenous population of macrophages (>96% F4/80^+^).

BMDM and TEPM were plated in 96-well tissue culture plates at 1x10^5^ cells/well for 16 h in complete RPMI at 37°C and 5% CO_2_ until complete adherence. After culture of TEPM, non-adherent contaminating peritoneal cells were eliminated by repeating three gentle washing of wells with pre-warmed culture medium or PBS. After stimulation with *Sc* BG compounds or dectin-1 ligands controls, supernatants from both cell types were collected and stored at -20°C until further use.

### Quantification of cytokines and chemokines by ELISA or multiplex EIA

Cytokines were quantified by a customized multiplex (Milliplex-MAP, Merck Millipore, France) assay kit and Luminex 100 IS instrument (Luminex, USA) at the Anexplo phenotyping service platform (CHU Rangueil, Toulouse, France). Thirteen cytokines were systematically assayed in BMDM: TNFα, IL-1β, IL-10, IL-6, CXCL1 (KC), CXCL2 (MIP-2) and CCL2 (MCP-1). Individual cytokine detection kits were also used to quantify mouse TNFα, IL-6 and IL-1β (Biolegend, Ozyme-France), and GM-CSF (R&D Systems, France).

### RT qPCR gene expression analysis

Total RNA was purified with the RNeasy Mini Kit (Qiagen, Hilden, Germany) according to the manufacturer’s instructions. RNA was quantified with a NanoDrop^®^1000 spectrophotometer and NanoDrop 1000.3.7 software and RNA quality was assessed using the Agilent RNA 6000 Nano Kit on BioAnalyzer and 2100 Expert Software (Agilent Technologies, Santa Clara, USA) at the GeT-TRiX platform (INRA, Toulouse, France). Total RNA (300 ng) was reverse transcribed using the SuperScript III First-Strand Synthesis Super Mix Kit (Invitrogen) following the manufacturer’s protocol. Quantitative PCR was performed with the Biomark HD System (Fluidigm, France) at the GeT-PlaGe genotyping service platform (INRA, Toulouse, France) according to the manufacturer’s recommendations. Primer3plus software was used to design the primers (Table in S1 Table) and housekeeping genes were selected with GeNorm Software. The abundance of mRNA of interest was normalized to that of S*dha*, *Rpl9* and *Hprt1* and relative expression was calculated using the 2^-ΔΔCt^ method.

### Detection of ROS by chemiluminescence

The production of ROS by primary cultured BMDM was measured by chemiluminescence upon stimulation. WT and *Clec7a*^-/-^ cells were handled as described above and seeded in 96-black chimney well culture plates in phenol red-free complete RPMI at 1x10^5^ cells/well in triplicate for 16 h before the experiment. Luminol powder (Sigma-Aldrich) was suspended in DMSO and then diluted 1000-fold in cell culture medium according to the supplier’s recommendations. Immediately before ROS were measured, supernatant was removed and Luminol and BG products were added simultaneously to each well. The chemiluminescence of each well was continuously acquired for 2 h post-stimulation in a Tecan Infinite 200 microplate reader. To assess the contribution of CR3 to ROS production, WT cells were preincubated with anti-CD11b (αCD11b) mAb (M1/70) or a control rat IgG2b mAb (Biolegend, Ozyme-France) for 1 h and were then stimulated with BG compounds.

### Isolation of bone marrow neutrophils and chemotaxis assay

Murine bone marrow neutrophils were freshly isolated from femurs and tibias with Percoll. Red blood cells were lysed by ACK and BM cells were resuspended in a 62% v/v gradient of Percoll in HBSS-Prep (constituted by Ca-Mg-free HBSS supplemented with 0.5% heat-inactivated FBS) and centrifuged for 30 min at 1000g. The cloudy pellet of neutrophils was washed twice in HBSS-Prep. Purity and viability were estimated by flow cytometry and were found to be >90% and > 85%, respectively. Neutrophils (5x10^5^cells/well) were seeded in the upper chambers of a 96-well transwell plate with a 3-μm pore size polycarbonate membrane (Corning, Lifescience, USA). The lower chamber contained supernatants collected from a culture of WT and *Clec7a*^-/-^ C57Bl/6 (and WT DBA/2 in S4A Fig) BMDM pretreated for 8 h with *Sc* BG compounds (100 μg/mL). After a 30 min incubation in a cell culture incubator (37°C, 5% CO_2_), the upper chambers were removed and the absolute number of migrated neutrophils was determined by flow cytometry using MACSQuant Analyzer.

### Assessment of phagocytosis and bactericidal activity

GFP-expressing mutants of the *S*. *aureus* HG001 strain were prepared as described previously by *Accarias et al* [21]. To assess the phagocytosis and bactericidal activity of BMDM, cells were first stimulated 8 h with 100 μg/mL of *Sc* CW compounds (BG15, BG65 and BG75) in 24-well plate (5x10^5^cells/well) as described below and then infected at an MOI = 10 with HG001-GFP bacteria for 1 h (37°C, 5% CO_2_). The supernatant was removed and cells were washed twice with warm PBS. Warm complete RPMI supplemented with Gentamicin^™^ was then added for 4 or 16 h. At the end of the incubation, cells were lysed with 0.1% Triton X-100 PBS and intracellular bacteria were labeled with propidium iodide (PI) (ImmunoChemistry Technologies, USA) according to the manufacturer’s instructions. Cell lysates were analyzed by flow cytometry (MACSQuant^®^, Miltenyi Biotech, Germany) and the amount of live and dead engulfed bacteria were determined using the GFP^+^PI^-^ and GFP^+^PI^+^ gates respectively (MACSQuantify^™^ Software). Data regarding phagocytosis and bactericidal activity are representative of two independent experiments (Data in S4C Fig).

### Statistical analysis

All experiments were performed three times unless otherwise specified and data are expressed as the mean ± SD of the values from all experiments. Each condition was performed in triplicate. Statistical significance was assessed using a two-tailed unpaired *Student’s t-test* with a threshold set at *p* < 0.05. Mean values shown with different letters on plots are significantly different.

## Results

### NFκB activity in murine macrophages varies according to the degree of BG enrichment

**Fig 1:**
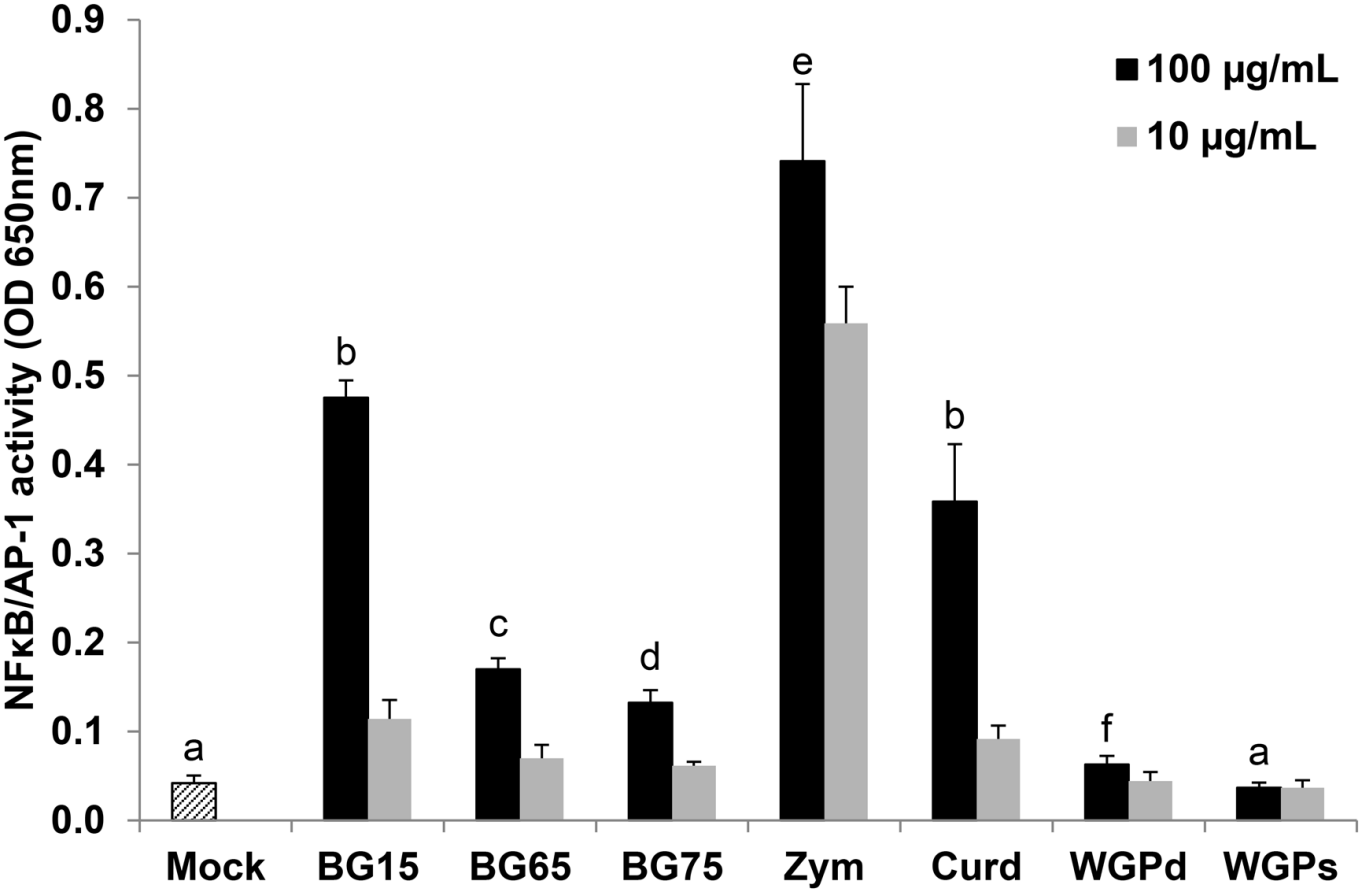
*Saccharomyces cerevisiae (Sc)* extracts enriched in β-glucan (BG) are much weaker inducers of NFκB/AP-1 activity in RAW-Blue^™^ macrophages than other sources of BG. NFκB/AP-1 activity was determined by a colorimetric enzyme assay using RAW-Blue^™^ macrophages as reporter cell line. Cells were treated for 16 h with 100 or 10 μg/mL of various *Sc* BG extracts (BG15, BG65 and BG75) or with zymosan, curdlan, dispersible WGPd and soluble WGPs as controls. Data are expressed as the mean OD 650nm ± SD of three independent experiments performed in triplicate. Mean values not sharing the same letter are significantly different according to the *Student’s t-test* (*p* < 0.05). Comparisons are shown for BG compounds used at 100 μg/mL.

We used RAW macrophage reporter cell line to examine the effect of two BG-enriched *Sc* CW extracts as compared to other BG sources on the NFκB pathway (Table 1). At a concentration of 100 μg/mL, *Sc* compounds enriched in BG (BG65 and BG75) were very much weaker inducers of NFκB/AP-1 activity than crude extract (BG15) or zymosan (Fig 1). Similarly, NFκB/AP-1 activity was low in RAW macrophages stimulated with a commercial source of purified β(1–3)(1–6)-glucan, WGPd as a control which confirms that at least two different sources of enriched BG are poor inducers of NFκB/AP-1 activity. Furthermore, soluble WGP from *Saccharomyces cerevisiae* failed to activate NFκB/AP-1 in these cells. Conversely, curdlan, the linear β(1,3)-glucan extracted from *Alcaligenes faecalis*, strongly activated NFκB/AP-1. Interestingly, in cells treated with a lower concentration of compounds (10 μg/mL), only zymosan and not BG15 and curdlan, was able to activate NFκB/AP-1, indicating that zymosan was more potent to stimulate NFκB and AP-1 pathway.

To confirm that highly-enriched BG do not activate NFκB/AP-1, we tested a large range of concentrations in reporter cells and found that higher concentrations of the enriched compounds BG65 and BG75 did not either stimulate NFκB/AP-1 activity (Data in S1 Fig), whereas, BG15 and zymosan induced NFκB/AP-1 activity to the same extent as 100 ng/mL of ultraPure LPS. Taken together, these data strongly suggest that BG alone cannot consistently activate NFκB/AP-1.

### *Sc* CW extracts enriched in BG activate dectin-1 but not TLR2/4

**Fig 2:**
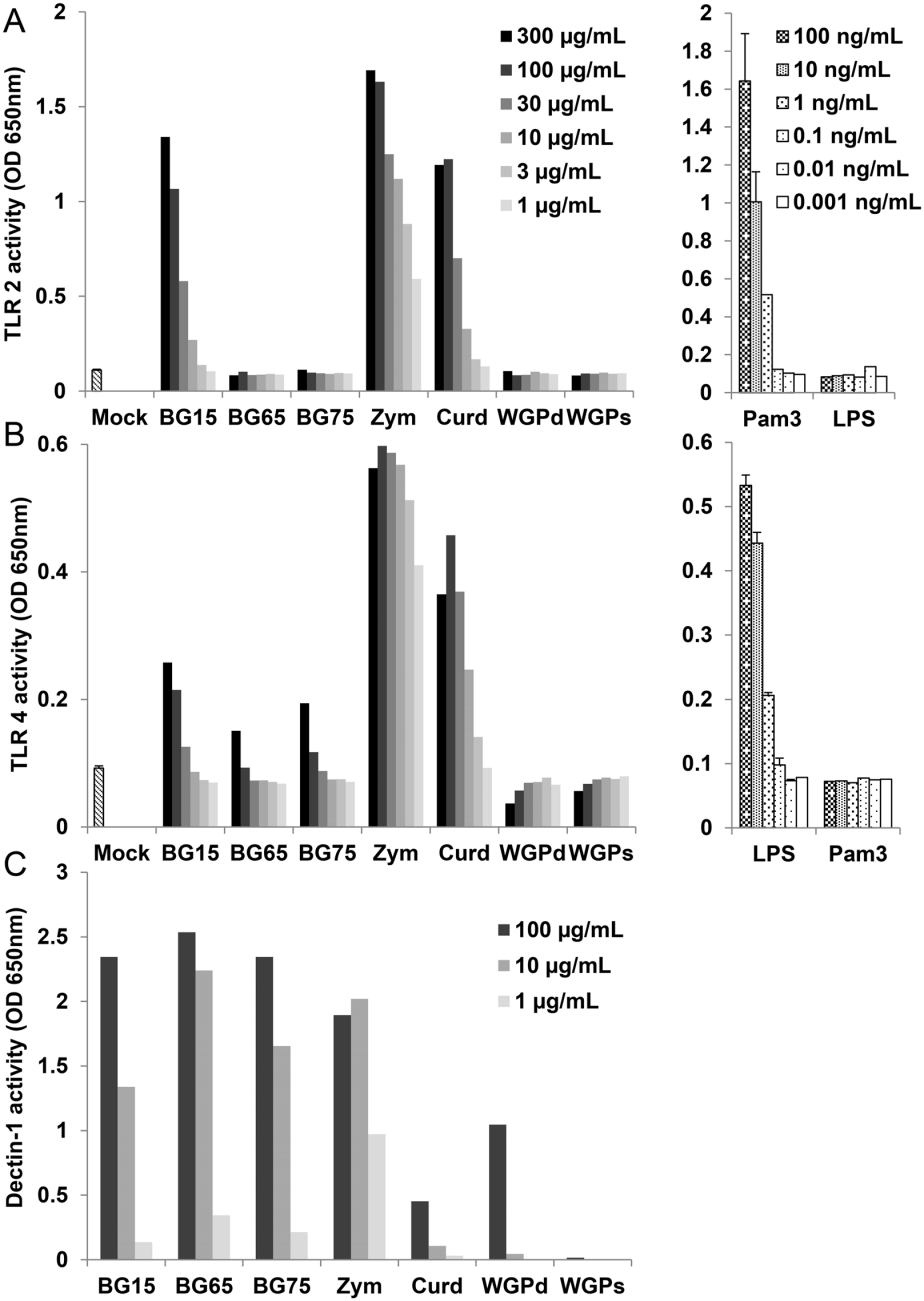
Enrichment of BG from yeast cell wall extracts strongly abolishes TLR2/4-related NFκB/AP-1 activities conversely to dectin-1. (A-C) HEK-Blue^™^-hTLR2, -hTLR4 and hDectin-1 cells were incubated with serially diluted *Sc* BG cell wall extracts (BG15, BG65 and BG75) or their BG controls (zymosan, curdlan, dispersible and soluble WGP) for 16 h in culture medium containing the reporter reagent (37°C, 5% CO_2)_. Each cell line was stimulated with a 10-fold serial dilution (from 100 ng/mL to 0.001 ng/mL) of control ligand, Pam3CSK4 for HEK-Blue^™^-hTLR2 and ultraPure LPS for HEK-Blue^™^-hTLR4, respectively as shown in the panels on the right of A and B. The NFκB/AP-1-related activity of TLR2, TLR4 and dectin-1 was assessed in supernatants by a colorimetric assay. The OD value of a blank control, which corresponds to the OD value of HEK-Blue detection medium, was subtracted from the OD values of samples. The results are presented as OD 650 nm values and are representative of three independent experiments.

Given that particulate BG-enriched *Sc* CW, but not crude extracts of *Sc* CW, failed to induce substantial NFκB/AP-1 activity, we next investigated whether these extracts stimulated the two main TLRs, using TLR2 and TLR4 reporter cell lines. Consistent with previous data, *Sc* crude extracts like zymosan and BG15, but also curdlan, activated TLR2 to a similar extent as 100 ng/mL of Pam3CSK4, a synthetic ligand of the TLR2 receptor (Fig 2A). Conversely, the BG-enriched extracts BG65 and BG75 as well as WGPd did not activate TLR2, suggesting that β(1–3)-glucans but not β(1–6)(1–3)-glucans interact with TLR2.

Similarly, the stimulation of HEK-Blue^™^-TLR4 cells with zymosan, curdlan, and to a lesser extent BG15, induced TLR4 signaling to a similar extent as 100 ng/mL of the positive control, ultraPure LPS; however, this was not true for highly-enriched BG compounds (BG65, BG75 and WGPd) (Fig 2B). Using similar assay with HEK-hTLR9 reporter cells, we completely rule out the presence of remaining DNA from yeast cells in crude BG15, zymosan, curdlan and enriched-BG compounds (S2 Fig). We next evaluated the ability of each compound to stimulate dectin-1 activity using the same approach. At the assayed concentrations (1–100 μg/mL), dectin-1 was activated by BG65, BG75 and to a lesser extent by BG15 and zymosan particles in a dose-dependent manner (Fig 2C). Conversely, dectin-1 activity was much lower in curdlan and WGPd, and soluble WGP did not activate dectin-1 at all. Strikingly, concentrations higher than 100 μg/mL for BG65 and BG75 provided saturated signals (data not shown). These data indicate that the purification process was efficient at removing the TLR2/4 activities and dectin-1 activity is correlated with the degree of BG enrichment.

### TNFα production in *Sc* CW products treated-macrophages correlates with their reported NFκB activity

**Fig 3:**
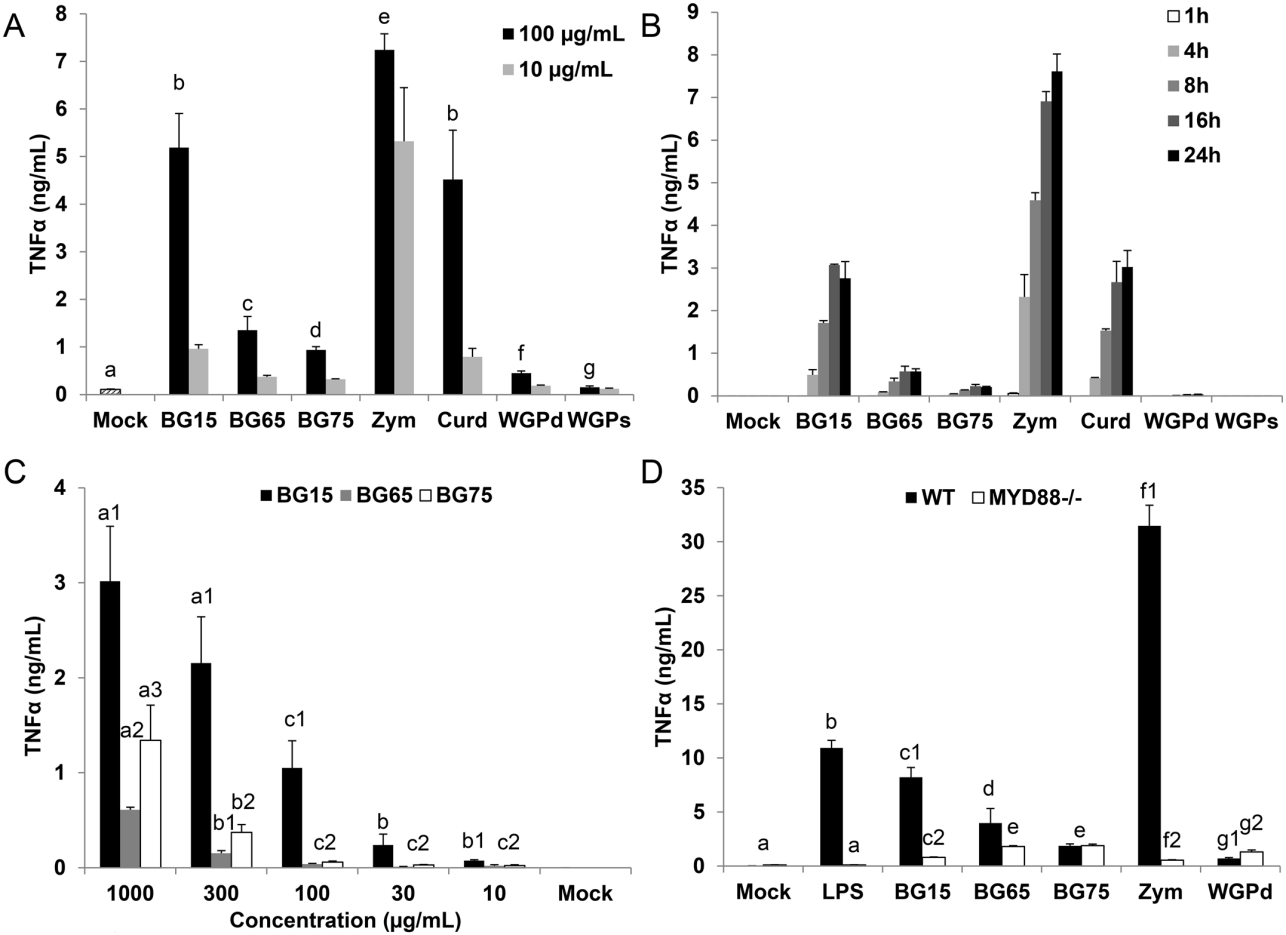
Murine macrophages treated with extracts enriched in BG produce less TNFα than those treated with unpurified *Sc* cell wall extracts. (A) RAW-Blue^™^ macrophages were treated with 100 or 10 μg/mL of *Sc* BG extracts (BG15, BG65 and BG75) or control (zymosan, curdlan, dispersible WGPd and soluble WGPs) for 16 h and TNFα was then quantified by ELISA. Comparisons are shown for BG compounds used at 100 μg/mL. (B-C) BMDM from C57Bl/6 mice were subcultured in 96-well plates. (B) Cells were stimulated for 1, 4, 8, 16 or 24 h with 100 μg/mL of *Sc* BG extracts (BG15, BG65 and BG75) or control (curdlan, dispersible WGPd and soluble WGPs). Supernatants were collected immediately after incubation. (C) Cells were incubated for 8 h with a 3-fold serial dilution from 1000 to 10 μg/mL of BG-purified compounds (BG15, BG65 and BG75). Supernatants were harvested immediately after incubation. (A-C) TNFα was measured using ELISA and the data are expressed as the mean ± SD of three independent experiments performed in triplicate. Mean values not sharing the same letter are significantly different according to the *Student’s test* (*p* < 0.05). (D) Immortalized BMDM (iBMDM) from WT and *MyD88*^-/-^ mice were subcultured in 96-well plates and stimulated for 8 h with 100 μg/mL of *Sc* BG extracts (BG15, BG65 and BG75) or control (zymosan and dispersible WGPd). LPS (100 ng/mL) was used as a positive control of MyD88 activation. Supernatants were harvested immediately after each incubation time to quantify TNFα by ELISA. Data are expressed as the mean ± SD of two independent experiments performed in triplicate. Mean values not sharing the same letter are significantly different according to the *Student’s t-test* (*p* < 0.05).

Our results suggest that NFκB/AP-1 activation in macrophages treated by *Sc* crude extracts or curdlan is directly related to the ability of these molecules to stimulate TLR2/4; therefore, we examined whether RAW-Blue^™^ macrophages also produce the NFκB-associated pro-inflammatory cytokine, TNFα. Consistent with the data presented above (Fig 1), TNFα production was highly correlated with NFκB activity. Indeed, only a small amount of TNFα (less than 1 ng/mL) was detected in the supernatant of cells treated with the highly-enriched *Sc* CW extracts, BG65, BG75 and WGPd, whereas at 100 μg/mL, BG15, zymosan and curdlan stimulate the production of more than 6 ng/mL of TNFα (Fig 3A). The 10-fold dilution of BG15, curdlan and to a lesser extent, zymosan, significantly diminished the cytokine production.

After having assessed the biological effects of *Sc* BG compounds on the RAW cell line, we next focused on primary murine macrophages (BMDM). We first performed a time-course assay to determine the optimal duration of exposure to BG compounds. BMDM produced lower amounts of TNFα than RAW-Blue^™^ cells, but the response to BG compounds was highly correlated between the two cellular models (Fig 3B). Moreover, this experiment confirmed that regardless of the incubation time, macrophages incubated with highly-enriched *Sc* BG compounds or WGP produce much lower amounts of TNFα than those stimulated with BG15, zymosan or curdlan (Fig 3B). Thus, cells incubated for a longer time with enriched compounds do not produce more cytokine. For all compounds, TNFα production began at 4 h and generally increased with time at a rate depending on the BG source. Zymosan triggered a strong, early and long-lasting response in macrophages which was higher than that of all other BG extracts tested. Indeed, the release of TNFα in response to *Sc* CW extracts enriched in BG peaked at 8–16 h of incubation and remained low until the end of the time course experiment, in contrast to crude *Sc* CW extracts (Fig 3B). Therefore, 8 h is the incubation time that leads to maximal cytokine production in response to *Sc* CW extracts. We then examined how the concentration of *Sc* BG influences TNFα production in BMDM (Fig 3C). At each concentration tested, more TNFα was released in response to BG15 than either BG65 or BG75 (from 2-fold higher at 1000 μg/mL to 15-fold higher at 100 μg/mL). Surprisingly, at high concentrations (300 and 1000 μg/mL), BG75 was more potent to induce TNFα production than BG65 whereas no difference was evidenced between these two BG-enriched compounds at lower concentrations tested (from 100 to 10 μg/mL). The largest difference in the response of BMDM to BG15 and BG75 occurred at a concentration of 100 μg/mL; therefore we used this concentration in the rest of the study. Given that crude *Sc* CW extracts strongly activated TLR2 and TLR4, we used *MyD88*-deficient macrophages to examine the involvement of the *MyD88-*signaling TLRs in TNFα production (Fig 3D). WT iBMDM produced more TNFα than RAW-Blue^™^ cells or BMDM, but the response to each compound was highly correlated between the cell types (Fig 3B and 3C) and as expected, the amounts of TNFα produced in WT iBMDM was inversely correlated with BG content. Indeed, WT iBMDM treated with zymosan, BG15 and LPS, produced large amounts of TNFα, in contrast to those treated with WGPd or *Sc* CW extracts enriched in BG (BG65 and BG75). The high production of TNFα induced by zymosan, BG15 and the positive control LPS was significantly impaired, and even absent in *MyD88*-deficient iBMDM. Furthermore, loss of *MyD88* had a little or no effect on the response of cells to BG65 or BG75, which further suggests that the activation of these cells by crude extracts of *Sc* CW mainly depends on TLR pathways. Altogether, these results show that increasing the purity of BG in *Sc* CW extracts impairs the production of cytokines by macrophages.

### Surface dectin-1 expression and the capacity of response to BG

**Fig 4:**
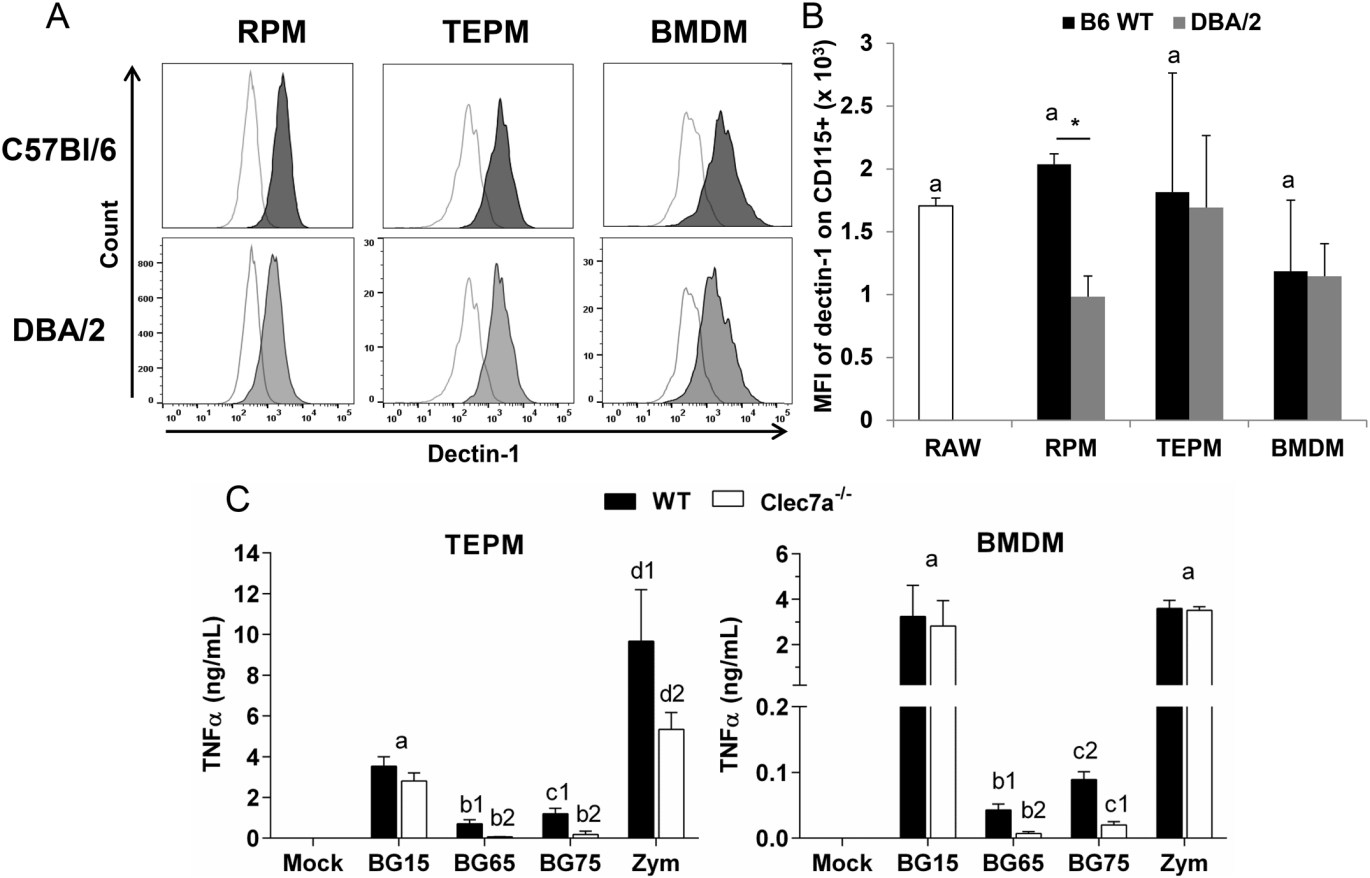
Murine primary macrophages including peritoneal macrophages and BMDM express similar levels of the BG receptor dectin-1 and exhibit the same ability to respond to *Sc* BG extracts. (A-B) Freshly isolated 4-days thioglycollate-elicited peritoneal macrophages (TEPM), resident peritoneal macrophages (RPM) and BMDM from WT and *Clec7a*^*-/-*^ C57Bl/6, and DBA/2 mice, and the macrophages cell line RAW-Blue^™^, used as control, were assessed for surface expression of dectin-1 by flow cytometry (MACSQuant^®^, Miltenyi Biotech, Germany). Doublets of cells were excluded at the beginning of the acquisition and a 7-AAD staining was used to discriminate death cells. The surface expression of dectin-1 was determined for each type of primary macrophages on CD115^+^ cells. (A) Data are expressed as the frequency of dectin-1^+^ among CD115^+^ cells. (B) The mean of fluorescence intensity (MFI) of dectin-1 was established on CD115^+^ cells. Results are presented as the mean ± SD MFI of dectin-1 corrected by either the MFI of isotype control for RAW macrophages, or the MFI of *Clec7a*^*-/-*^ C57Bl/6 for WT C57Bl/6 or DBA/2 primary macrophages. (C) Remaining TEPM and BMDM from WT and *Clec7a*^*-/-*^ C57Bl/6 mice isolated from these experiments were subcultured and stimulated with 100 μg/mL of crude or enriched *Sc* BG CW extracts (BG15, BG65 or BG75) or zymosan for 8 h (37°C, 5% CO_2_). Supernatants were collected immediately after incubation and were assessed for TNFα production by ELISA. Results are shown as the mean ± SD. All data are representative of three mice per group from three independent experiments performed in triplicate. *p < 0.05 (two-tailed unpaired *Student’s t-test)*; mean values not sharing the same letter are significantly different.

It has been previously reported that BG does not signal in BMDM in contrast to peritoneal macrophages or dendritic cells due to a low dectin-1 expression and defaults in its signaling pathway [18]. We first compared the dectin-1 expression on the surface of various cell types including resident peritoneal macrophages (RPM), thioglycollate-elicited macrophages (TEPM) and BMDM, isolated from 3 murine genetic backgrounds (WT or *Clec7a*^*-/-*^ C57Bl/6 and DBA/2). Both freshly isolated RPM and TEPM, but also BMDM from WT C57Bl/6 and DBA/2 mice expressed high surface levels of dectin-1 in a comparison with C*lec7a*^*-/-*^ C57BL/6 as a control (Fig 4A).

Expression of endogenous dectin-1 was very similar between the Raw-Blue^™^ cell line macrophage and primary cells, as indicated by measurement of the mean fluorescence intensity (MFI) on CD115^+^ macrophages (Fig 4B). Furthermore, surface expression was slightly higher on RPM from C57Bl/6 than RPM from DBA/2 whereas no difference could be observed between the two backgrounds on other macrophage types.

We next compared the ability of macrophages from the different sources to respond to crude or enriched *Sc* BG compounds. Crude BG extracts BG15 and especially zymosan, induced higher TNFα release than did enriched BG65 or BG75 (Fig 4C). Dectin-1-deficiency affects the production of TNFα in both cell types although the difference is higher with zymosan, and with BG-enriched extracts, a 10-fold diminution was noted in TEPM and BMDM (for BG65, 708 versus 61 pg/mL for TEPM, and 40 versus 6 pg/mL for BMDM in WT and C*lec7a*^-/-^ cells, respectively). Similarly, for BG75, TNFα production was 1193 versus 178 pg/mL in TEPM, and 89 versus 20 pg/mL for BMDM in WT and *Clec7a*^-/-^ cells, respectively. These data confirm the above description of low TNFα production triggered by highly-enriched BG compounds whatever the macrophage cell type.

### BG-enriched *Sc* CW extracts stimulate the late Clec7a-dependent expression of CSF2

**Fig 5:**
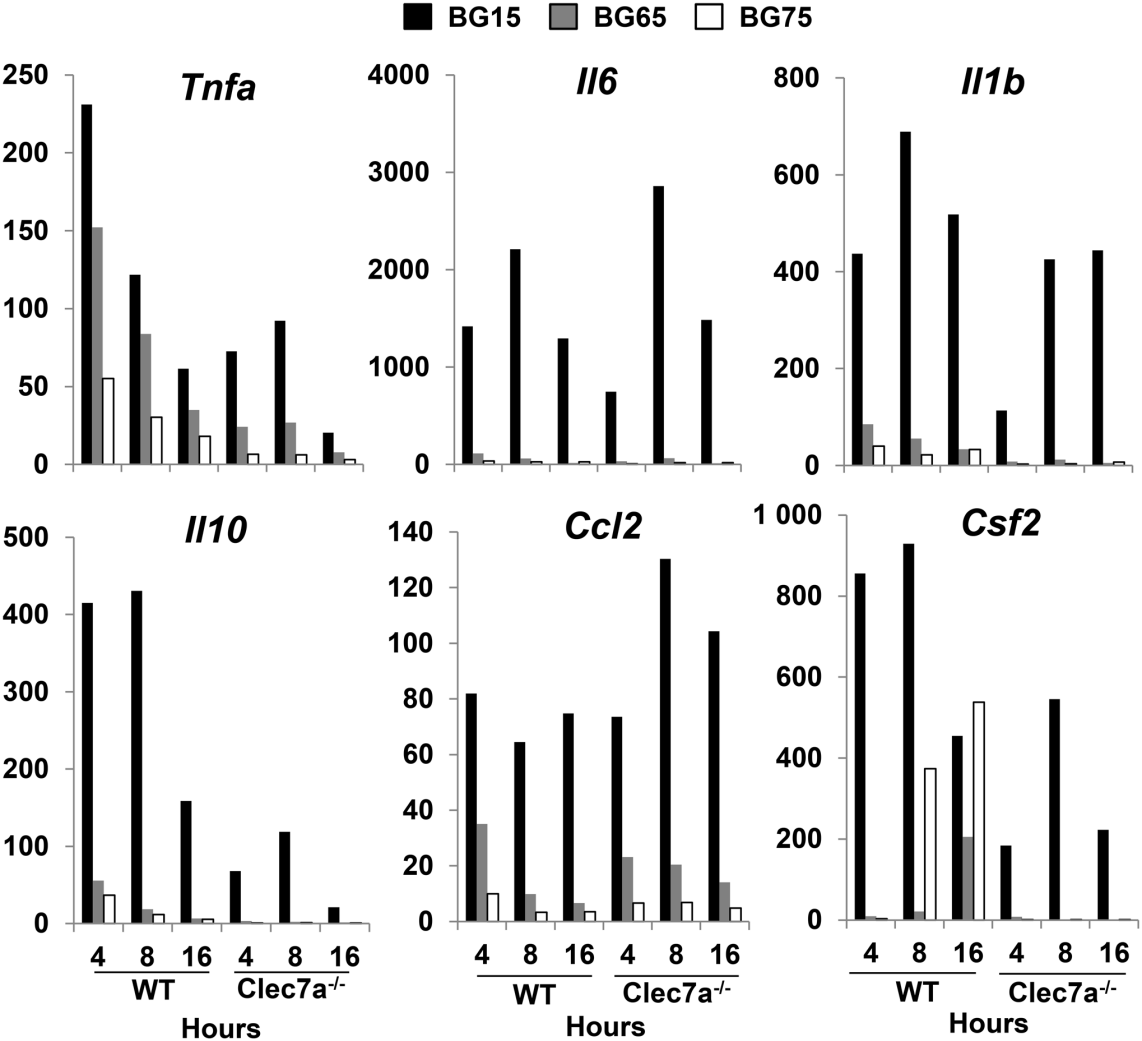
The timing of *Clec7a*-dependent *Csf2* expression depends on *Sc* BG content. WT and *Clec7a* (dectin-1)^-/-^ BMDM were stimulated with *Sc* BG extracts for 4, 8 or 16 h. After incubation, supernatants were removed and cells were lysed in RLT lysis buffer. Total RNA was extracted using the RNeasy Mini Kit and retro-transcribed with the SuperScript III First-Strand Synthesis Super Mix Kit. The expression of cytokine and chemokine genes was measured by quantitative PCR and normalized with three housekeeping genes (*Sdha*, *Rpl9* and *Hprt1*) selected with GeNorm Software. Individual quantitative PCR was performed on the LightCycler^®^ 480 system (Roche, Switzerland) using Power SYBR Green PCR Master Mix (Applied Biosystems) to confirm the results obtained with the Biomark method. Data are expressed as fold change related to the mock value, representative of two experiments performed in triplicate.

We next performed a high-throughput qPCR experiment to investigate the differential effects of crude and BG-enriched *Sc* CW extracts on dectin-1 pathway in BMDM. We analyzed the expression of several genes in WT and *Clec7a*^-/-^ BMDM stimulated with *Sc* BG compounds for different amounts of time. As expected, in both genetic backgrounds, BG15 induced the early and strong upregulation of pro-inflammatory but also anti-inflammatory genes (Fig 5). The expression of cytokine genes such as *Il1b*, *Il6* and *Il10* peaked between 4 and 8 h post-stimulation and then decreased until 16 h post-stimulation, whereas that of *Tnfa* was maximal from 4 h post-treatment. Surprisingly, *Tnfa* was upregulated by *Sc* BG-enriched compounds, albeit to a lesser extent than by BG15. The monocyte-attractant chemokine *Ccl2* was upregulated at 4 h post-incubation and its expression was maintained until 16 h in WT BMDM incubated with BG15 but decreased in cells treated with BG65 or BG75, showing that the expression kinetics varied depending on the gene products. Interestingly, BG-dependent gene expression was severely impaired in *Clec7a*^-/-^ BMDM. Indeed, the early induction of *Il10*, *Il6*, *Tnfa*, *Il1b* and *Csf2* gene expression observed in WT macrophages was partially (BG15) or mainly abrogated (BG65 and BG75) in *Clec7a*^-/-^ cells. Intriguingly, the kinetics of *Tnfa*, *Il1b*, and *Ccl2* gene expression differed between WT and *Clec7a*^*-/-*^ cells. The expression of *Tnfa* and *Il1b* was delayed in *Clec7a*^-/-^ cells treated with BG15, and at 8 h the expression of these genes was lower in *Clec7a*^-/-^ than in WT cells. The expression of*Ccl2* was also delayed in *Clec7a*^-/-^ macrophages treated with BG15, but this gene was still expressed at 16 h post-incubation and was even higher in deficient cells. Interestingly, the expression of *Csf2* in WT and *Clec7a*^-/-^ cells treated with BG15 resembled that of *Il10 and Il6*. Conversely, BG75 and to a lesser extent BG65, stimulated the late expression of *Csf2* that was completely absent in *Clec7a*^-/-^ cells. Taken together, these data demonstrate that in WT macrophages, the crude *Sc* CW extract BG15 triggers an earlier and stronger response than the BG-enriched *Sc* CW extracts BG65 and BG75 and, that dectin-1 is required to initiate the late upregulation of *Csf2* by BG-enriched compounds. Furthermore, the response of *Clec7a*^-/-^ cells to BG15 was mainly slower and weaker than that of WT cells, indicating that dectin-1 improves the early inflammatory response in cells stimulated with BG-containing *Sc* CW extracts.

**Fig 6:**
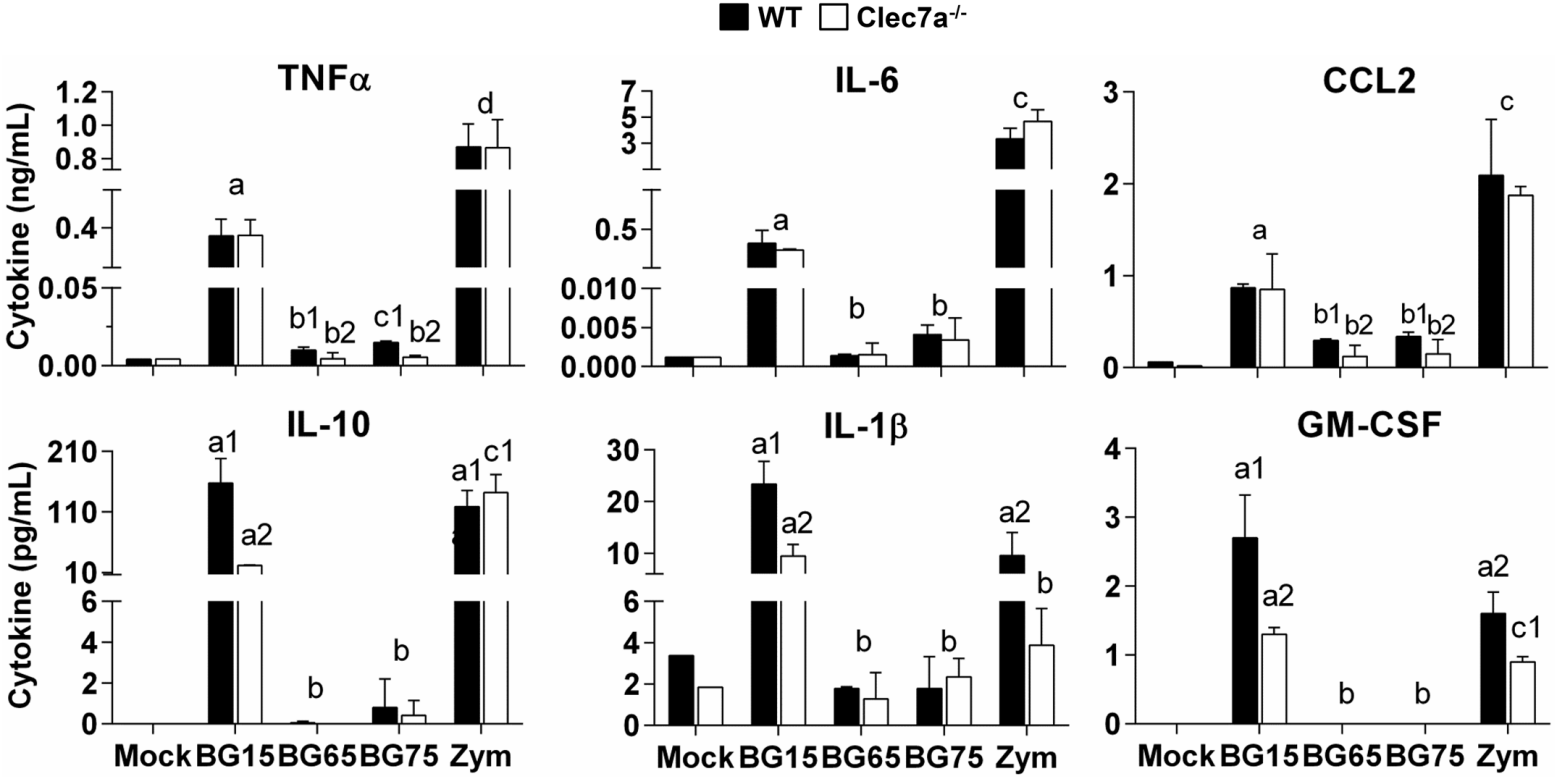
*Sc* cell wall crude products- and zymosan-induced cytokine productions are mainly dectin-1-dependent. WT and *Clec7a*^-/-^ BMDM were stimulated with *Sc* extracts enriched in BG for 8 h. After incubation, cell culture supernatants were harvested and stored at -20°C. Chemokines and cytokines (TNFα, IL-6, CCL2, IL-10 and IL-1β) were quantified using a customized multiplex assay and GM-CSF was measured with a cytokine detection kit. Data are expressed as the mean ± SD of three independent experiments performed in triplicate. Mean values not sharing the same letter are significantly different according to the *Student’s t-test* (*p* < 0.05).

We also assessed the secretion of cytokines in the supernatants from WT and *Clec7a*^-/-^ BMDM incubated with crude or BG-enriched *Sc* CW extracts. Consistent with the gene expression profiles of WT BMDM, the crude *Sc* CW extract, BG15, and zymosan significantly induced the production of all cytokines and chemokines examined (Fig 6). In contrast, the BG-enriched *Sc* CW extracts, BG65 and BG75, did not significantly stimulate the secretion of these cytokines and chemokines. We also incubated WT BMDM with *Sc* CW extracts for longer than 8 h. Although levels of GM-CSF in BG65 and BG75-treated cells were higher at 16 and 24 h post-treatment, they remained lower than those triggered by zymosan or BG15 (Data in S3 Fig). We also performed similar experiments in *Clec7a*^-/-^ macrophages to investigate the role of the dectin-1 pathway. TNFα, IL-6 and CCL2 productions were similar in WT and *Clec7a*^-/-^ BMDM stimulated with BG15 or zymosan, but the secretion of IL-1β, IL-10 and GM-CSF was significantly lower in *Clec7a*^-/-^ cells. Similarly, although WT macrophages treated with the enriched *Sc* BG compounds, BG65 and BG75 produced very low amounts of cytokines and chemokines, the production of TNFα and CCL2 was significantly lower in *Clec7a*^-/-^ than in WT cells. No statistical differences were found for IL-6, IL-10 and IL-1β comparing *Clec7a*^-/-^ and WT cells response regarding BG65 and BG75 incubation. Altogether, these data suggest that the interaction between BG and dectin-1 has low stimulatory effects when the crude *Sc* CW extract BG15 and zymosan are used. These results further suggest that *Sc* CW enriched in BG extracts poorly activate cytokine production in macrophages.

### *Sc* BG-enriched compounds poorly induce neutrophil recruitment via dectin-1

**Fig 7:**
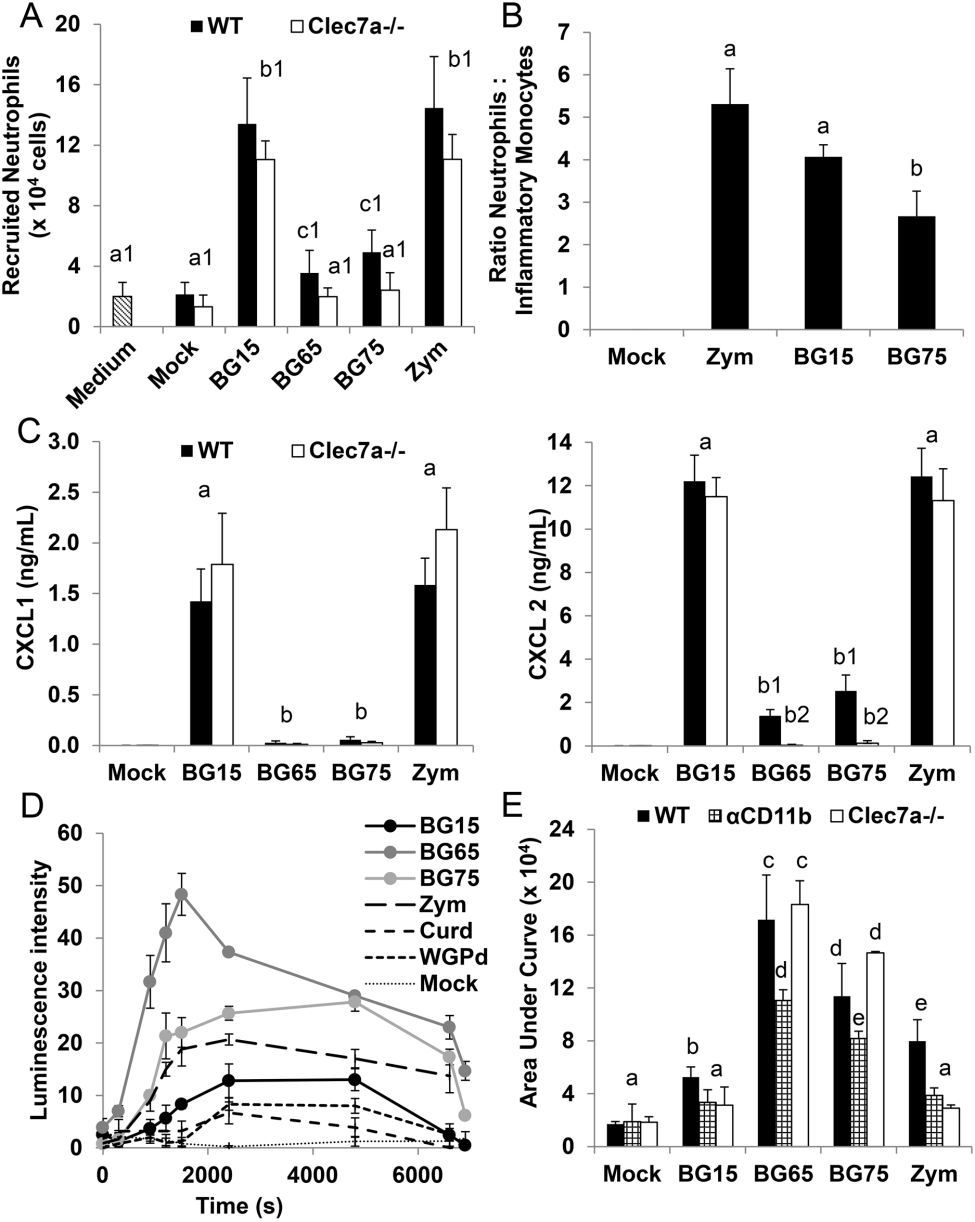
*Sc* BG-enriched extracts poorly induce neutrophil recruitment via dectin-1 but strongly stimulate ROS production via CR3 and not dectin-1. (A) Transwell chemotaxis assay to assess the chemotactic activity of supernatants from WT and *Clec7a*^-/-^ BMDM stimulated with *Sc* BG extracts (BG15, BG65 and BG75) for 8 h. BM neutrophils of WT C57Bl/6 mice (5x10^5^cells/well) were incubated for 30 min at 37°C with the supernatants and the neutrophils migrating into the lower chamber of the 96-well transwell plate were quantified by flow cytometry with an absolute counting system. Control wells containing medium (RPMI supplemented with 10% heat-inactivated FBS) were included in the chemotaxis assay. Data are expressed as the mean ± SD of three independent experiments performed in triplicate. (B) C57Bl/6 mice (n = 4/group) were challenged ip with 100 μg of BG15, BG75 or zymosan under 500 μL of PBS. After 6 h of incubation, peritoneal cells were collected and the number of recruited neutrophils and inflammatory monocytes were assessed by flow cytometry as described in Material and Methods section. Data are presented as the mean ± SD of four individual mice measurements and are representative of two independent experiments. (C) WT and *Clec7a*^*-/-*^ BMDM were stimulated with *Sc* BG extracts for 8 h. After incubation, cell culture supernatants were collected and stored at -20°C. CXCL1 and CXCL2 were quantified using a customized multiplex assay. Data are expressed as the mean ± SD of three independent experiments performed in triplicate. (A-C) Mean values not sharing the same letter are significantly different according to the *Student’s test* (*p* < 0.05). (C-D) WT (D-E) and *Clec7a*^-/-^ BMDM (E) were subcultured in 96-well plates and incubated simultaneously with Luminol and 100 μg/mL of *Sc* BG extracts (BG15, BG65 and BG75) or control (zymosan, curdlan, dispersible WGPd and soluble WGPs). ROS production was assessed immediately from the intensity of luminescence in each well, which was measured every 5 min for 2 h with a Tecan plate reader. (D) Results are expressed as the mean of luminescence intensity ± SD of two independent experiments performed in triplicate. (E) WT BMDM were incubated for 1 h with LEAF^™^ anti-mouse CD11b (αCD11b) or with its rat control isotype IgG2a mAb and were then treated with BG products. Data were recorded into Prism 6 (GraphPad Software, San Diego, CA) and analyzed by the area under curve (AUC) method. Levels of ROS produced by αIgG2a-WT BMDM were comparable to those of untreated cells. Results are expressed as the mean of three independent experiments performed in triplicate. Mean values not sharing the same letter are significantly different according to the *Student’s t-test* (*p* < 0.05).

We found that the various BG compounds tested influence the activation status of macrophages, which probably affects the inflammatory response, and in particular, cell recruitment. To further characterize the effects of *Sc* CW compounds on the other components of the innate immune system, we first evaluated the chemotactic properties of culture supernatants from BMDM treated with *Sc* CW compounds. Consistent with our previous results, culture supernatants from cells treated with the crude extract BG15 or with zymosan were significantly more chemo-attractant than their counterparts obtained from cells treated with the BG-enriched *Sc* CW extracts, BG65 and BG75 (Fig 7A). BG75- and BG65-conditioned medium were slightly more chemoattractant than mock control or medium alone, with a non-significant upward tendency for BG75- compared to BG65-conditioned medium. We used *Clec7a*^-/-^ cells to investigate the involvement of dectin-1 in this process. Dectin-1-deficiency severely altered the chemotactic activity of BG75-conditioned medium, which had the same effect on *Clec7a*^-/-^ cells as BG65-conditioned medium, the mock control or medium alone. Conversely, dectin-1-deficiency poorly influenced the chemotactic properties of BG15- or zymosan-conditioned medium, suggesting that the production of chemo-attractants by crude BG15 and zymosan does not depend on dectin-1 signaling.

To confirm these findings, we also performed the chemotaxis assay with supernatants of BMDM differentiated from DBA/2 mouse bone-marrow precursors because DBA/2 expresses full-length dectin-1 whereas the C57Bl/6 expresses a truncated splice variant [24,25]. Supernatants from DBA/2 cells treated with BG15 or BG65 recruited significantly more neutrophils than those from C57Bl/6 cells treated with these compounds whereas the migration of neutrophils was similar in response to supernatants derived from C57Bl/6 and DBA/2 cells treated with BG75 (Data in S4A Fig).

Furthermore, we assessed the severity of the peritoneal inflammation using the cell number ratio between recruited neutrophils and inflammatory monocytes in a peritonitis model after BG15, BG75 or zymosan challenge, as recently reported by *Lastrucci et al* [26]. For both zymosan and BG15, the cell recruitment was significantly dominated by neutrophils (p < 0.01) which attested to an intense inflammatory reaction (Fig 7B). In contrast, BG75 intra-peritoneal injection induced much lower neutrophil infiltration. These observations are clearly consistent with our *in vitro* results showing that crude BG15 and zymosan are strong inducers of inflammation in macrophages.

To confirm these results, we quantified the two major chemokines involved in neutrophil recruitment, CXCL1 (KC) and CXCL2 (MIP-2) in culture supernatants of macrophages stimulated *in vitro* by *Sc* BG compounds. Consistent with our previous observations, BMDM treated with the crude *Sc* CW extract BG15 or zymosan produced higher amounts of these chemokines than those treated with the BG-enriched compounds, BG65 and BG75 (Fig 7C). Again, BG75 tended to induce higher amounts of chemokine and recruit slightly more cells than BG65-conditioned medium. Intriguingly, dectin-1-deficiency did not significantly influence chemokine production in cells treated with BG15, but strongly impaired CXCL2 by BMDM treated with BG-enriched *Sc* CW compounds. Together, these results further suggest that crude products promote neutrophil recruitment independently of BG-dectin-1 interactions, in contrast with BG-enriched *Sc* CW extracts which poorly induce neutrophil recruitment via dectin-1.

### *Sc* BG-enriched compounds strongly stimulate ROS production via CR3 but not dectin-1

We next investigate the capacity of BG extracts to modulate phagocytosis and killing, notably by producing ROS. Strikingly, ROS production within the first 30 min post-stimulation was significantly higher in WT BMDM treated with BG65 and BG75 than in those treated with crude BG15 and zymosan (Fig 7D). Conversely, curdlan and WGPd prompted macrophages to release very low amounts of ROS, similar to the levels detected in mock conditions. To determine whether the response to *Sc* BG compounds is mediated by the dectin-1 or the CR3 pathways, we performed the same experiments in *Clec7a*^-/-^ BMDM and with a neutralizing antibody against CR3 (αCD11b). Surprisingly, ROS production was similar in *Clec7a*^-/-^ and WT BMDM treated with BG65 and tended to be higher (+30%) in response to BG75 in *Clec7a*^-/-^ than in WT BMDM (Fig 7E). In contrast, ROS production in response to BG15 was consistently 40% lower in *Clec7a*^-/-^ than in WT BMDM and was 64% lower in zymosan-treated *Clec7a*^-/-^ BMDM than their zymosan-treated WT counterparts. Other BG-containing compounds such as curdlan and WGPd induced ROS production neither in WT nor *Clec7a*^-/-^ cells (Data in S4B Fig). Inhibition of the CR3 pathway in BMDM with the blocking monoclonal antibody (clone M1/5) led to a moderate to severe impairment of ROS production in cells treated with BG-containing *Sc* CW extracts (Fig 7E). CR3 blockade impaired zymosan-stimulated and BG15-stimulated ROS production by 52% and about 40%, respectively. Interestingly, blocking the CR3 pathway in BG65- and BG75-stimulated BMDM moderately inhibited ROS production (by approximatively 30%). Thus, the BG-enriched *Sc* CW extracts BG65 and BG75 strongly stimulate ROS production partly through the CR3 but independently of the dectin-1 pathway. Conversely, crude *Sc* CW extracts stimulate low but dectin-1- and CR3-dependent ROS production, despite their TLR2/4 activity. To confirm these results, we analyzed the ability of BMDM pretreated with *Sc* BG compounds to phagocyte and kill bacteria. Incubation with BG-enriched or crude *Sc* CW extracts did not appear to influence the ability of BMDM to phagocyte the *S*. *aureus* HG001 strain bacteria (Data in S4C Fig). However, even if no statistical differences were evidenced, macrophages incubated with BG65 or BG75 tended to kill more bacteria than those incubated with BG15 or mock. Thus, these findings, and those of our ROS experiments, suggest that *Sc* CW extracts enriched in BG stimulate the bactericidal activity of macrophages.

## Discussion

In this study, we show that BG are not sufficient by themselves to trigger cytokine and chemokine production in various types of murine macrophages, despite promoting other activities such as ROS production and bacterial killing.

Our results suggest that dectin-1 alone is not sufficient to trigger NFκB/AP-1 signaling in macrophages but crosstalk between dectin-1 and other PRRs, such as TLR2 and TLR4, greatly enhances NFκB-associated cytokine production, notably when TLR signals are weak. BG make up around 50% of the *Saccharomyces cerevisiae* CW [16], and as shown here, *Sc* CW extracts contains a mixture of various ligands that activate other PRRs on immune cells. Upon enrichment, these PRR signals are partially or totally removed, leaving dectin-1 ligands more less intact.

Many studies have described the immuno-modulatory effects of ‘β-glucans’. However, contradictory data resulting from these studies may lead to misconceptions about biological activities of BG. These discrepancies may be explained by the heterogeneity of BG preparations, especially regarding source, degree of purity and molecular structure. For example, even the exact composition of ‘zymosan’ remains unclear because zymosan may differ from a provider to another, is rarely handled as supplied and may have undergone treatment before use, such as depleted zymosan that is used as a selective ligand of Dectin-1. Consequently, several properties have been unfairly ascribed to BG, which prompted us to use a triad of *Sc* CW extracts increasingly enriched in BG.

In our study, the incubation of macrophages with extracts from the same strain, substantially altered cell activation depending on the BG content. Our cell reporter assays showed that BG65 and BG75 lowly activate macrophages, notably for cytokine production, and are deprived of MyD88-related activity, despite strong dectin-1 activity. Consistently, we also showed that activation of dectin-1 poorly stimulated NFκB-associated TNFα production. Furthermore, *Sc* CW extracts enriched in BG, especially BG75, induced the production of small but similar amounts of TNFα in WT and *MyD88*^-/-^ cells. These results seem to contrast with those of previous studies involving particulate BG, but are consistent with those showing that soluble BG has no effect on macrophages and dendritic cells [2]. Indeed, particulate BG-containing extracts, including curdlan and other *Sc-* or *Candida albicans* CW extracts, and even a synthetic ‘BG’ [27], has been shown to stimulate cells via the NFκB pathway, resulting in the production of large amounts of TNFα, IL-12, -6, -10, CCL2, and MIP-2 via a dectin-1-dependent pathway [11,13,18,28,29]. *In vivo* studies also suggest that BG initiate the mobilization of immune cells (neutrophils) to the blood and other organs, like the spleen, by strongly promoting the release of cytokines and chemokines [30]. In the context of wound healing, *Sc* BG also activate NFκB via TLR2 and dectin-1 in murine and human macrophages *in vitro* and *in vivo*, and recruit macrophages to the site of healing [31]. Thus, particulate BG-containing *Sc* CW extracts are generally thought to promote strong cellular responses. However, some studies report conflicting findings. For example, *Shah et al* found that *Sc* BG does not upregulate cytokine production (IL-1β, IL-2, IL-6, IL-10, TNFα) in microglia cells [32], which is consistent with the results of our study. Of note, RNA values were in lines with cytokine production for all cytokines, although slight unmeaning variation can be observed for TNFα in BG15 condition between WT and *Clec7a*^-/-^ BMDM, that could result from post-transcriptional regulation in WT cells, a mechanism previously described by several authors for this cytokine [33]. Our data were also in lines with results previously observed using depleted zymosan, showing weak IL-10 production and IL12p40 induction compared to zymosan [19,34]. This seems to suggest that BG-enriched extracts could be alike to depleted zymosan, a hot alkali-treated zymosan that activates dectin-1 but not TLR2.

Furthermore, particulate BG products have often been poorly characterized (in terms of structure, composition and source), which complicate the comparison of the results. Indeed, *Adams et al* found that the interaction of BG with dectin-1 strongly depends on the primary structure of BG, including its chain length and side-chain branching [7], such that structurally different BG may have distinct biological effects on immune cells. Furthermore, activation of dectin-1 has been shown to require the formation of a ‘phagocytic synapse’ on the cell membrane [17]. In addition to contain observable particles under microscope, all BG suspensions used in this study (except the soluble WGPs), induced a substantial response in our dectin-1 reporter assay, even stronger than the positive control WGPd. Consistently with findings of *Goodridge et al*, this strongly suggests that our particulate BG extracts were able to trigger dectin-1 clustering. Interestingly, some authors reported that BMDM from C57BL/6 poorly express dectin-1 and this was the cause of their low responsiveness following highly-enriched BG stimulation as comparison to peritoneal cells or bone marrow-derived dendritic cells. It might be true that according to the method of *in vitro* maturation, BMDM could express significantly different levels of the main BG receptor [35]. Indeed, *Gersuk et al* reported that cells incubated in L929 medium, that we used in this study, exhibit increased expression of surface dectin-1 compared to cells matured in recombinant M-CSF. Moreover, expression of dectin-1 on BMDM surface was observed by other authors [12,18]. Furthermore, we also assessed that BMDM, RPM, TEPM and the RAW-Blue^™^ cells expressed similar levels of this receptor, meaning that there is no direct correlation between dectin-1 surface expression levels and the ability of macrophages to respond to enriched-BG. This was clearly elucidated by the lower amounts of TNFα production in TEPM stimulated by BG65 and BG75 compared to BG15 and zymosan, but also in RAW cells which express high endogenous levels of dectin-1 and exhibit low NFκB activity after BG-enriched treatment. TEPM isolated from DBA/2 also released low TNFα in response to BG65 and BG75 (data not shown), even if that mouse strain express a more active isoform of dectin-1 compared to C57Bl/6 strain [24,25]. Thus, we demonstrated that macrophages from various sources were unable to produce a robust inflammatory response following dectin-1 signaling mediated by BG-enriched treatment. This is in contradiction with the conclusion of *Goodridge et al* saying that responsiveness of BMDM from C57Bl/6 following BG binding was due to expression of additional factor which restrict dectin-1/CARD9 signaling for TNFα induction [18]. This is not taking into account the fact that BMDM are globally low responsive cells compared to resting or elicited peritoneal macrophages.

We also observed that in macrophages treated with *Sc* CW extracts enriched in BG, GM-CSF was not secreted at an early stage of the response. Instead, the expression of *Csf2* (which encodes GM-CSF) was upregulated from a late time-point according to the BG-content of extracts in WT cells that was totally abrogated in their *Clec7a*^-/-^ counterparts. It has been reported that the production of GM-CSF upon stimulation with BG-enriched *Sc* CW extracts also promotes dectin-1 expression at the surface of activated cells [18,36,37,38]. Although we observed a slight overexpression of dectin-1 induced by BG-enriched compounds (data not shown), the secretion of GM-CSF was a late consequence of dectin-1 triggering, suggesting that BG stimulate the expression of *Csf2* indirectly, by a secondary mechanism, possibly a feedback loop that remains to be deciphered.

The non-pathogenic yeast CW contains mannans, chitins and glycoproteins, as is the case for zymosan [14] and BG15. Therefore, it is important to consider the role of these molecules in the cellular responses as they can interact with other PRRs or even collaborate with dectin-1 [39,40]. For example, according to their structure, mannans are recognized by MR (Mannose Receptor), dectin-2 or even TLR4, resulting in cytokine production [6,41]. This may explain why the activity BG65 was slightly higher than that of BG75, as BG65 contains almost 4% of mannans in contrast to BG75 which mannan-content remains less than 1%. This is especially true for BG15 or zymosan that induced the release of pro-inflammatory cytokines and chemokines (TNFα, IL-6, IL-1β, CCL2) but also the anti-inflammatory cytokine, IL-10. Furthermore, crude or poorly enriched *Sc* CW extracts have strong TLR2 and in a much lesser extent TLR4 activity, although their ability to activate dectin-1 resembles that of BG-enriched *Sc* CW extracts, indicating that the inflammatory response elicited by these crude compounds is not mediated by dectin-1 alone. *Gantner et al* [19] and several other studies have reported that zymosan activates macrophages [10,32,37,42,43]. This activity has been attributed to BG but it probably results from other cell wall components, as suggested by our experiments and also depleted zymosan versus zymosan comparisons in the literature, or from cooperative or synergistic signals. Indeed, dectin-1 and TLR2 work together to control the cellular response to microbes [29,44]. Both dectin-1 and TLR stimulate the production of TNFα and IL-12 via NFκB in response to particulate stimuli containing zymosan, suggesting that dectin-1 is an important partner of TLR2 on macrophages. Furthermore, BMDM or BMDC from *MyD88*- or TLR2^-/-^ mice produce almost no TNFα, IL-6, -10 and -23 in response to zymosan [10,45,46], suggesting that TLR pathways are required to trigger the pro-inflammatory response to crude *Sc* CW BG-containing extracts like zymosan. We also found that *MyD88*-deficiency strongly affected the ability of zymosan and BG15 to induce the production of TNFα. However, some authors conclude that dectin-1 is required to elicit an inflammatory response upon zymosan stimulation. Indeed, the overexpression of dectin-1 in RAW264.7 cells enhances TNFα and IL12p40 secretion in response to zymosan [10,19], and *Taylor et al* showed that dectin-1-deficient cells fail to release TNFα following stimulation with opsonized or unopsonized zymosan [11]. In our study, we were unable to make similar conclusions regarding the response of *Clec7a*^-/-^ BMDM to zymosan, although we observed a slightly altered production of TNFα by *Clec7a*^-/-^ TEPM receiving zymosan. Furthermore, we found that dectin-1 deficiency did not affect the production of pro-inflammatory cytokines/chemokines in response to zymosan in BMDM, except for that of GM-CSF, IL-10 and IL-1β, an inflammasome-related cytokine which secretion was reported to be mediated through dectin-1-dependent pathway in human macrophages [47,48]. It is not surprising that IL-10 production induced by zymosan was severely impaired in *Clec7a*^-/-^ BMDM, as far as *Elcombe et al* previously described the ERK- and p38 MAPK- CREB pathways involvement leading to IL-10 release after dectin-1 engagement [34]. Our observations are also consistent with the results of *Shah et al* who reported the same effect after neutralizing dectin-1 on microglia cells [32].

We also evidenced that zymosan induced TLR4 activity in a reporter cell line, which may explain why zymosan strongly activated macrophages. The effects of BG-containing *Sc* CW extracts on TLR4 activity have been rarely investigated or verified upon treatment. However, a few studies report that TLR4 contributes somewhat to the effects of dectin-1 on immune cells [6,29,42,43]. Chemokine production induced by zymosan or zymosan-like extracts induces the recruitment of cells, such as monocytes via CCL2 and also neutrophils mainly via CXCL1 (KC) and CXCL2 (MIP2). Our findings suggest that these events occur in a dectin-1-independent-manner. Furthermore, the macrophage inflammatory protein MIP2 is produced in large amounts by RAW264.7 macrophages and dendritic cells stimulated with curdlan [36,49]. Surprisingly, for all BG-containing *Sc* CW extracts tested, supernatant derived from DBA/2 cells was more chemotactic than that derived from C57Bl/6 cells, in accordance with the fact that the full length form of dectin-1 is more effective than the truncated form.

ROS production was significantly higher in BMDM treated with BG65 and BG75 than those treated with crude *Sc* CW extracts. ROS production was not affected by the loss of *Clec7a*, suggesting that ROS production depends on BG but is not mediated by dectin-1. However, poorly enriched compounds such as BG15 and zymosan stimulate the production of low amounts of ROS production in part via dectin-1. These findings contrast with previous studies, showing that zymosan induces strong respiratory burst in human and murine macrophages [17,19,50,51], consistent with our findings, some studies showed that macrophages produce only low amounts of ROS in response to zymosan [11] and respiratory burst induced by zymosan is severely impaired following the genetic deletion of dectin-1 [11,50,51]. Furthermore, ROS promote the induction of IL-10 in human macrophages treated with zymosan [52]. We also observed that zymosan and BG15 stimulate IL-10 expression by BMDM in a dectin-1-dependent manner (confirmed at protein levels), which is consistent with the findings of *Elcombe et al* [34]. Conversely, BG-enriched *Sc* CW extracts triggered the production of significant amounts of ROS but negligible amounts of IL-10 mRNA and protein, both in WT and *Clec7a*^-/-^ BMDM. This suggests that ROS production depends on BG but not dectin-1, and raises doubt about the link between IL-10 and ROS production. Instead, neutralization of CD11b (CR3) impaired ROS production by at least 30% in macrophages incubated with BG-enriched *Sc* CW compounds, and to an even greater extent in those incubated with crude *Sc* CW compounds. Thus, CR3 partially initiates respiratory burst following the recognition of unopsonized BG compounds. These findings are consistent with several studies demonstrating that CR3 is involved in recognition and phagocytosis of nonopsonized zymosan, leading to superoxide production [53]. Furthermore, CR3 appears to be the most important receptor for phagocytosis, although dectin-1 is necessary for the optimal uptake of BG [54]. This may explain why we observed that ROS production was similar between WT and *Clec7a*^-/-^ BMDM treated with BG-enriched *Sc* CW extracts. However, we found that the inhibition of CR3 was not able to impair ROS production to levels observed with crude *Sc* CW compounds. This suggests that other(s) receptor(s) is involved in respiratory burst such as the TLR/MYD88 pathway, although *Gantner et al* demonstrated that this pathway is not linked to ROS production [19]. The phagocytic and bactericidal activity of BMDM treated *Sc* CW compounds were consistent with the amount of ROS that they produced. Consequently, BG are of great interest not only for anti-fungal, but also for anti-bacterial immunity. Indeed, *Clec7a*- deficient hosts are more susceptible to *C*. *albicans* infections than their WT counterparts [11]. This is consistent with the fact that BG from the pathogenic yeast *Candida albicans* are shielded from dectin-1 by outer cell wall components, allowing the pathogen to evade host immune responses [6,28,55,56].

## Conclusion

In conclusion, our findings demonstrate that the recognition of *Sc* CW BG-enriched extracts by macrophages relies on C-type lectins receptors among which dectin-1 appears central. However, in absence of a concomitant TLR stimulation, the triggering of dectin-1 pathway results in a weak NFκB response and consequently the production of low amounts of pro- and anti-inflammatory cytokines. Conversely, these BG-enriched extracts are able to activate macrophages for strong respiratory burst. Despite the intense research led in this field, the events that occur following the recognition of *Sc* BG compounds by dectin-1 and other PRRs remain unclear, probably because the compounds used until now have been too poorly characterized. Considering our findings, highly BG-enriched *Sc* CW extracts may be useful in the future to determine the mechanism of action of BG to evaluate their utility as immuno-modulatory molecules.

## Supporting Information

S1 Fig *Sc* cell wall crude extract (1 mg/mL) induces the same level of NFκB activity as the optimal dose of LPS in RAW-Blue^™^ macrophages. RAW-Blue^™^ macrophages were pretreated with 10-fold serial dilutions from 1 mg/mL to 1 ng/mL of *Sc* BG extracts (BG15, BG65 and BG75) or with 100 ng/mL of ultraPure LPS for 16 h. NFκB activity was assessed by reading OD at 650nm after another 16 h incubation of cell culture supernatants with Quanti-Blue^™^ reagent. Data are expressed as the mean ± SD of OD at 650nm minus the background value. Two independent experiments were performed in duplicate.
(PDF)

S2 Fig *Sc* cell wall extracts and curdlan are deprived of TLR9 activity. HEK-Blue^™^-hTLR9 were incubated with serially diluted *Sc* BG cell wall extracts (BG15, BG65 and BG75) or their BG controls (zymosan and curdlan) for 16 h in culture medium containing the reporter reagent (37°C, 5% CO_2)_. This cell line was stimulated with a 3-fold serial dilution (from 1 to 0.01 μg/mL) of control ligand, CpG ODN2006 as shown in the right panel. The NFκB/AP-1-related activity of TLR9 was assessed in supernatants by a colorimetric assay. The OD value of a blank control, which corresponds to the OD value of HEK-Blue detection medium, was subtracted from the OD values of samples. The results are presented as OD 650 nm values and are representative of three independent measurements.
(PDF)

S3 Fig GM-CSF production by BMDM is time- and BG-dependent. WT BMDM were stimulated with *Sc* extracts enriched in BG for 8, 16 or 24 h. After incubation, cell culture supernatants were harvested and stored at -20°C. GM-CSF was measured with a cytokine detection kit provided by R&D Systems (France). Data are expressed as the mean ± SD of three independent experiments performed in triplicate. Mean values not sharing the same letter are significantly different according to the *Student’s t-test* (*p* < 0.05).
(PDF)

S4 Fig *Sc* BG-enriched extracts poorly induce neutrophil recruitment via dectin-1 and strongly promote bactericidal activity in a BG- but not dectin-1-dependent manner. (A) Transwell chemotaxis assay to assess the chemotactic activity of supernatants of WT and *Clec7a*^-/-^ BMDM from C57Bl/6 mice and WT DBA/2 BMDM stimulated with *Sc* BG extracts (BG15, BG65 and BG75) for 8 h. Bone marrow neutrophils of WT C57Bl/6 mice were incubated for 30 min with the supernatants and the neutrophils migrating into the lower chamber of the transwell plate were quantified by flow cytometry with an absolute counting system. Data are expressed as the mean ± SD of three independent experiments performed in triplicate. Mean values not sharing the same letter are significantly different according to the *Student’s t-test* (*p* < 0.05). (B) *Clec7a*^-/-^BMDM were subcultured in 96-well plates and incubated simultaneously with Luminol and 100 μg/mL of *Sc* BG extracts (BG15, BG65 and BG75) or control (zymosan, curdlan, dispersible WGPd and soluble WGPs). ROS production was assessed immediately from the intensity of luminescence in each well, which was measured every 5 min for 2 h with a Tecan plate reader. Results are expressed as the mean ± SD of one experiment performed in triplicate and are representative of two independent experiments. (C) The bactericidal activity of BMDM (5x10^5^cells/well in 24-well plate) primed with 100 μg/mL of *Sc* BG extracts (BG15, BG65 and BG75) for 8 h was measured after infection with a GFP- expressing mutant of *S*. *aureus* HG001 strain at MOI 10 for 1 h, as described in the *Material and Methods*. Cells were washed, lysed in 0.1% Triton X-100 PBS and intracellular bacteria were labeled with propidium iodide (PI). Cell lysates were analyzed by flow cytometry (MACSQuant^®^, Miltenyi Biotech, Germany) and the amount of live and dead engulfed bacteria were determined using the GFP^+^PI^-^ and GFP^+^PI^+^ gates, respectively (MACSQuantify^™^ Software). A control sample without Gentamicin^™^ was included in this assay to estimate the number of bacteria engulfed by BMDM. Data are expressed as the mean ± SEM of two independent experiments performed in duplicate. The panel on the right shows the percentage of live bacteria phagocytized at 4 and 16 h post-challenge compared to 1 h of bacteria-cells interaction.
(PDF)

S1 Table Primers designed for analysis of gene expression in BMDM by quantitative PCR. (PDF)
