# Supplementary figures and images for: Triggering Dectin-1-Pathway Alone Is Not Sufficient to Induce Cytokine Production by Murine Macrophages
Source: PLoS One. 2016 Feb 3;11(2):e0148464. doi: 10.1371/journal.pone.0148464 (PMC4739705; doi:10.1371/journal.pone.0148464)

**S1 Fig**

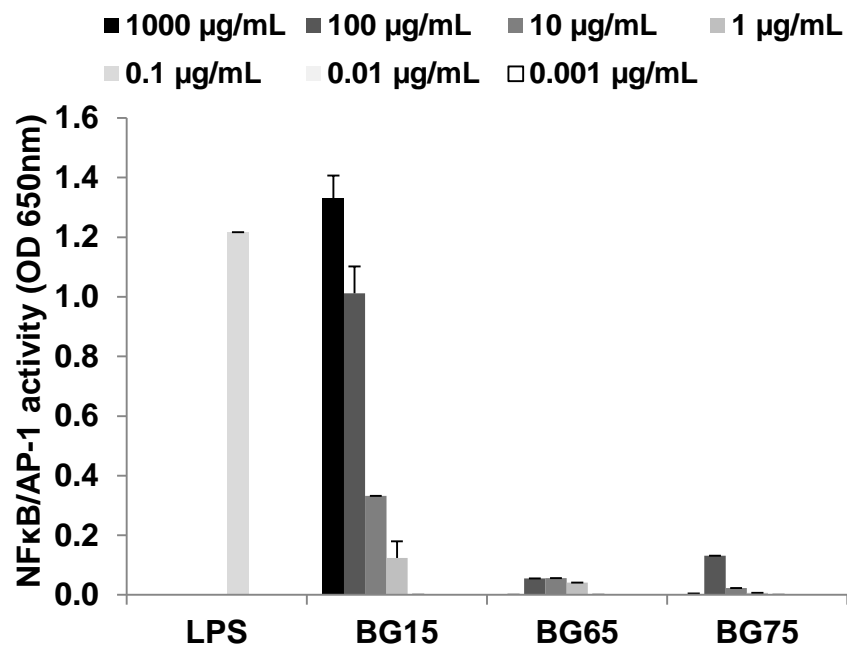

Supplement: S1 Fig — RAW-Blue™ macrophages were pretreated with 10-fold serial dilutions from 1 mg/mL to 1 ng/mL of Sc BG extracts (BG15, BG65 and BG75) or with 100 ng/mL of ultraPure LPS for 16 h. NFκB activity was assessed by reading OD at 650nm after another 16 h incubation of cell culture supernatants with Quanti-Blue™ reagent. Data are expressed as the mean ± SD of OD at 650nm minus the background value. Two independent experiments were performed in duplicate. (PDF) [file pone.0148464.s001.pdf]

**S2 Fig**

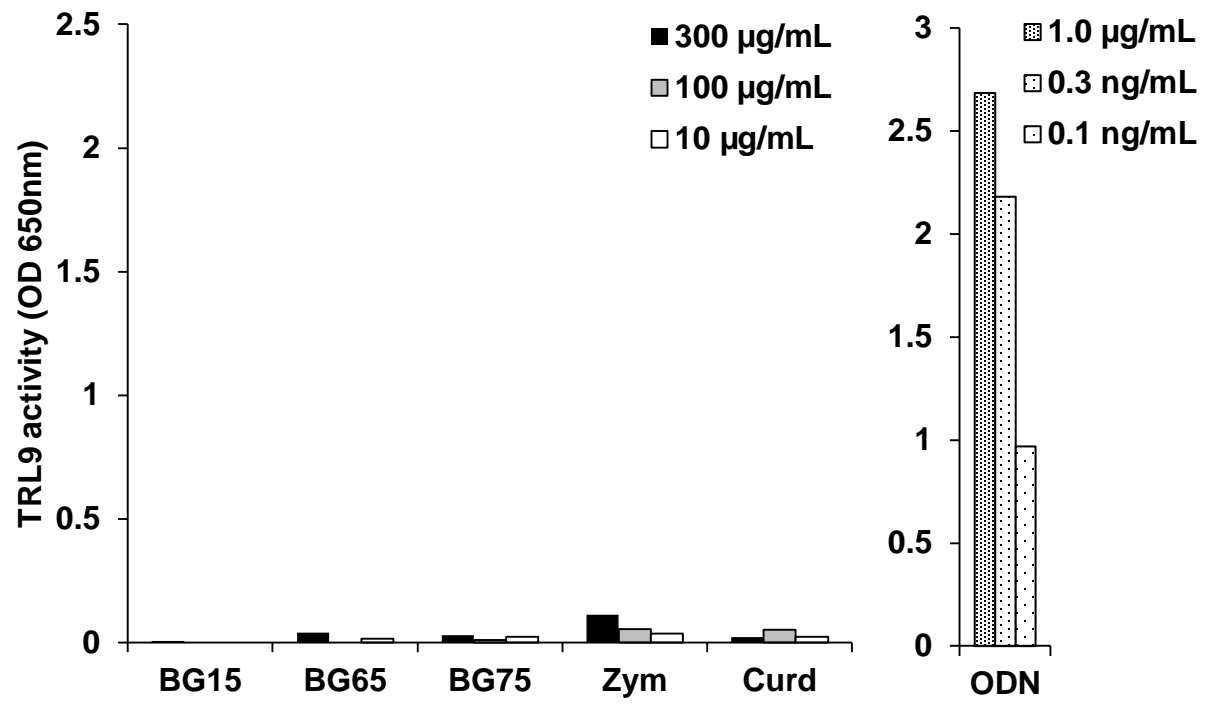

Supplement: S2 Fig — HEK-Blue™-hTLR9 were incubated with serially diluted Sc BG cell wall extracts (BG15, BG65 and BG75) or their BG controls (zymosan and curdlan) for 16 h in culture medium containing the reporter reagent (37°C, 5% CO2). This cell line was stimulated with a 3-fold serial dilution (from 1 to 0.01 μg/mL) of control ligand, CpG ODN2006 as shown in the right panel. The NFκB/AP-1-related activity of TLR9 was assessed in supernatants by a colorimetric assay. The OD value of a blank control, which corresponds to the OD value of HEK-Blue detection medium, was subtracted from the OD values of samples. The results are presented as OD 650 nm values and are representative of three independent measurements. (PDF) [file pone.0148464.s002.pdf]

**S3 Fig**

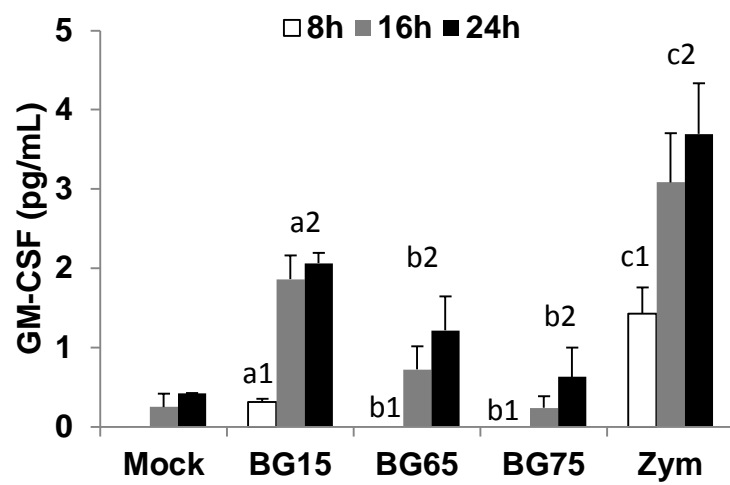

Supplement: S3 Fig — WT BMDM were stimulated with Sc extracts enriched in BG for 8, 16 or 24 h. After incubation, cell culture supernatants were harvested and stored at -20°C. GM-CSF was measured with a cytokine detection kit provided by R&D Systems (France). Data are expressed as the mean ± SD of three independent experiments performed in triplicate. Mean values not sharing the same letter are significantly different according to the Student’s t-test (p < 0.05). (PDF) [file pone.0148464.s003.pdf]

**S4 Fig**

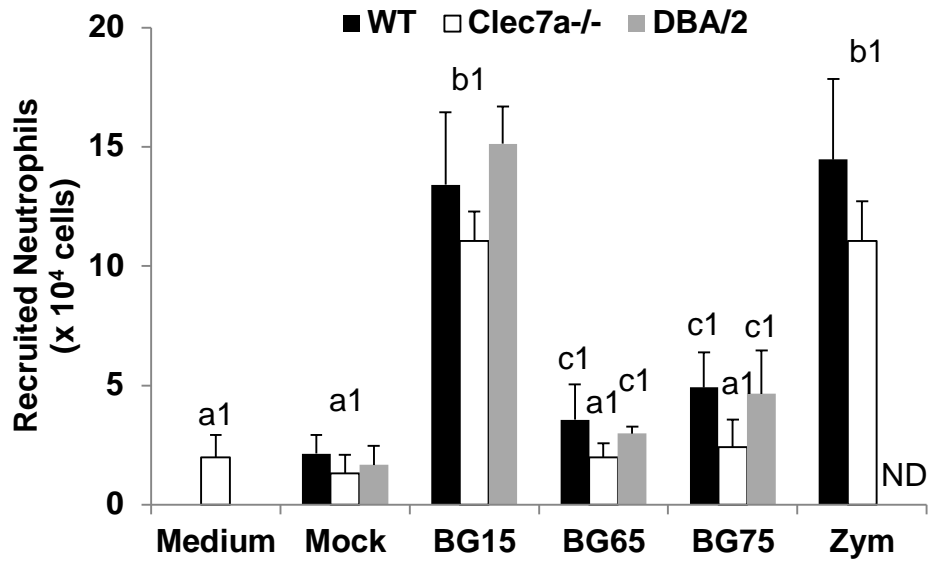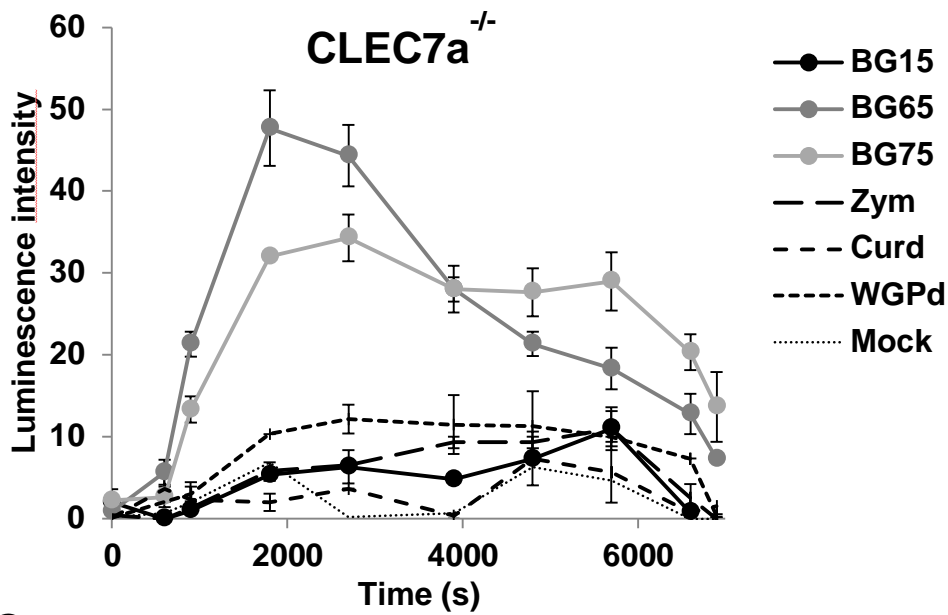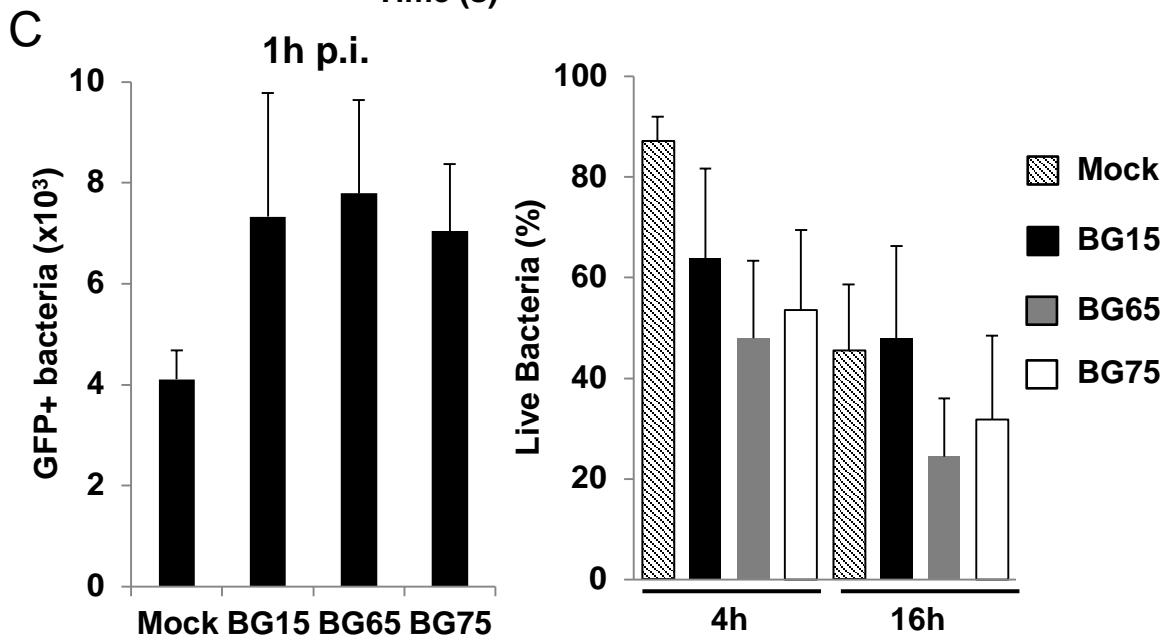

Supplement: S4 Fig — (A) Transwell chemotaxis assay to assess the chemotactic activity of supernatants of WT and Clec7a-/- BMDM from C57Bl/6 mice and WT DBA/2 BMDM stimulated with Sc BG extracts (BG15, BG65 and BG75) for 8 h. Bone marrow neutrophils of WT C57Bl/6 mice were incubated for 30 min with the supernatants and the neutrophils migrating into the lower chamber of the transwell plate were quantified by flow cytometry with an absolute counting system. Data are expressed as the mean ± SD of three independent experiments performed in triplicate. Mean values not sharing the same letter are significantly different according to the Student’s t-test (p < 0.05). (B) Clec7a-/-BMDM were subcultured in 96-well plates and incubated simultaneously with Luminol and 100 μg/mL of Sc BG extracts (BG15, BG65 and BG75) or control (zymosan, curdlan, dispersible WGPd and soluble WGPs). ROS production was assessed immediately from the intensity of luminescence in each well, which was measured every 5 min for 2 h with a Tecan plate reader. Results are expressed as the mean ± SD of one experiment performed in triplicate and are representative of two independent experiments. (C) The bactericidal activity of BMDM (5x10^5^cells/well in 24-well plate) primed with 100 μg/mL of Sc BG extracts (BG15, BG65 and BG75) for 8 h was measured after infection with a GFP- expressing mutant of S. aureus HG001 strain at MOI 10 for 1 h, as described in the Material and Methods. Cells were washed, lysed in 0.1% Triton X-100 PBS and intracellular bacteria were labeled with propidium iodide (PI). Cell lysates were analyzed by flow cytometry (MACSQuant®, Miltenyi Biotech, Germany) and the amount of live and dead engulfed bacteria were determined using the GFP+PI- and GFP+PI+ gates, respectively (MACSQuantify™ Software). A control sample without Gentamicin™ was included in this assay to estimate the number of bacteria engulfed by BMDM. Data are expressed as the mean ± SEM of two independent experiments performed in duplicate. The [file pone.0148464.s004.pdf]
