# Supplementary material for: Triggering Dectin-1-Pathway Alone Is Not Sufficient to Induce Cytokine Production by Murine Macrophages
Source: PLoS One. 2016 Feb 3;11(2):e0148464. doi: 10.1371/journal.pone.0148464 (PMC4739705; doi:10.1371/journal.pone.0148464)
Supplement: S1 Table — (PDF) [file pone.0148464.s005.pdf]

	Primers (5' -> 3')	
**Genes**	**Forward**	**Reverse**
TNFa	CATCTTCTCAAAATTCGAGTGACAA	TGGGAGTAGACAAGGTACAACCC
Il6	GAGGATACCACTCCCAACAGACC	AAGTGCATCATCGTTGTTCATACA
Il1b	CAACCAACAAGTGATATTCTCCATG	GATCCACACTCTCCAGCTGCA
Il10	GGTTGCCAAGCCTTATCGGA	ACCTGCTCCACTGCCTTGCT
Ccl2	CTTCTGGGCCTGCTGTTCA	CCAGCCTACTCATTGGGATCA
Csf2	CCGTAGACCCTGCTCGAATA	TGCCTGTCACATTGAATGAA
Sdha	ATTGTGCCTGGTCTGTATGC	AATTTGCTCCAAGCCGGTTG
Rpl9	TGGTCCCTGCTCTCAAG	GGCCTTTTCCTTCCGTTTCTC
Hprt1	AGGACTGAAAGACTTGCTCGAG	AATCCAGCAGGTCAGCAAAG
